# Pan-cancer analysis of COL15A1: an immunological and prognostic biomarker

**DOI:** 10.1007/s12672-024-01200-z

**Published:** 2024-08-01

**Authors:** Lei Zhu, Qianheng Jiang, Jun Meng, Haichun Zhao, Jie Lin

**Affiliations:** 1 Graduate School, Dalian Medical University, Dalian, Liaoning China; 2 Department of General Surgery, Panjin Liao-Oil Field Gem Flower Hospital, Panjin, Liaoning China; 3 Department of General Surgery, Liaoning Provincial Cancer Hospital, Shenyang, Liaoning China; 4 School of Stomatology, China Medical University, Shenyang, Liaoning China

**Keywords:** COL15A1, Immune infiltration, TMB, MSI, Prognosis

## Abstract

Collagen, type XV, alpha 1 (COL15A1) belongs to the collagen superfamily, which can influence disease progression by modulating immune pathways. Although the growing number of investigations demonstrating the indispensable role of COL15A1 in the progression of certain tumors, no pan-cancer assessment of COL15A1 is accessible to date. Therefore, the available data was used to explore the role of COL15A1 in 33 types of tumors and to investigate their potential immune function. Numerous bioinformatics approaches were used to research the potential oncogenic role of COL15A1, including analysis of tumor prognosis, microsatellite instability (MSI), tumor mutational burden (TMB), single nucleotide polymorphism (SNP), drug sensitivity, immune cell infiltration, and the correlation between cancer stem cells (CSCs) and COL15A1 expression. The outcome implies that most tumors had a high expression of COL15A1, and COL15A1 manifested different relationships with prognosis in different tumors, including both positive and negative correlations. COL15A1 was also found to have a significant correlation with MSI, TMB, and immune infiltrating cells. Our study suggests that COL15A1 may serve as a prognostic marker for malignancy because of its differential expression in tissues and their function in tumor immunity.

## Introduction

Tumors are known for their high mortality rates and poor prognosis. Despite the enormous advances in medical technology over the last few years, no absolute cures have been found for tumors as yet [1]. With extensive research on immunotherapy, scientists have found that immune checkpoint blockade therapy might be a superior tumor treatment [2]. We can use various databases to carry out pan-cancer gene expression analysis and evaluate the correlations between gene expression and clinical prognosis, which may help us discover new immunotherapy targets since public databases (including TCGA and GTEx) have been constantly updated and developed in recent years [3].

Collagen, type XV, alpha 1 (COL15A1) is a member of the collagen superfamily that can influence the development of certain disorders by promoting angiogenesis and participating in the immune response [4, 5]. For example, COL15A1 causes the formation of liver cirrhosis by triggering angiogenesis [4]. Recently, mounting evidence has shown that COL15A1 also had an impact on tumor growth and invasion [6]. Several reports of COL15A1 being related to the occurrence of liver cancer [7], ovarian cancer [8], lung cancer [6], and rectal cancer [9] have been documented in the literature.

However, prior research on the involvement of COL15A1 in cancers has been focused on individual tumor types rather than a pan-cancer examination of the relationship between COL15A1 and diverse tumors. As a result, we evaluate the differences in COL15A1 expression levels in diverse malignancies and their link with tumor prognosis using several databases such as TCGA, cBioPortal, Human Protein Atlas (HPA), and Genotype Tissue-Expression (GTEx). Furthermore, we analyzed the potential relation between COL15A1 expression levels and tumor prognosis in 33 types of tumors from MSI, TMB, immune infiltration levels, and GSEA enrichment assessment. To examine the biological roles of COL15A1 in tumors, we looked at the relationship between COL15A1 and cancer fibroblasts, and drug sensitivity. Our findings suggest that COL15A1 may be a predictive factor for cancer and that tumor-infiltrating immune cells, TMB, and MSI may influence COL15A1’s role in tumor immunity.

## Methods

### Data analysis

Transcriptome data and clinical information are downloaded from TCGA (Widely recognized as the most informative database on cancer) and UCSC (An online database for exploring gene expression and clinical data, http://xena.ucsc.edu/). Download gene expression data of different tissues in GTEx (A database for analyzing gene expression in different tissues and cells). We used Strawberry Perl to process the downloaded data to obtain COL15A1 gene expression data, and organized them into a data matrix for subsequent analysis.

### Differential expression analysis of COL15A1

Matched standard and tumor samples were compared using the downloaded data, and tumors were processed according to COL15A1 expression levels in 33 tumor types in descending order. The expression data for these tumor types were Log2 transformed, and a two-group t-test was conducted, with p < 0.05 deemed a notable discrepancy in expression between normal and tumor tissues. In addition, R software (version 4.1.0) was used to analyze the data, and the R program "ggplot2" was used to create box plots.

### Immunohistochemical staining

We selected tumors with significant differential expression of COL15A1 gene by GEPIA2 (http://gepia2.cancer-pku.cn/), including liver hepatocellular carcinoma (LIHC), kidney renal clear cell carcinoma (KIRC), cervical squamous cell carcinoma and endocervical adenocarcinoma (CESC), ovarian serous cystadenocarcinoma (OV), pancreatic adenocarcinoma (PAAD), stomach adenocarcinoma (STAD) Immunohistochemical imaging of COL15A1 protein expression in six normal and tumor tissues were downloaded and processed via HPA (https://www.proteinatlas.org/), an online immunohistochemical analysis tool, to further investigate changes in COL15A1 expression at the protein level.

### The relationships between COL15A1 expression and clinical phenotype

Clinical phenotype and prognosis data were downloaded from UCSC. Then, four indicators were selected to evaluate the patient’s prognosis, namely; disease-specific survival (DSS), overall survival (OS), progression-free interval (PFI), and disease-free interval (DFI). Kaplan–Meier (KM) approach and log-rank assessment were employed for survival analysis (p < 0.05) for individual cancer types. To obtain the curves of survival, use the R packages “survival” and “survminer.” Cox assessment, which might evaluate the pan-cancer association between COL15A1 expression and survival, was also carried out employing the R packages “survival” and “forest plot.”

To explore their connection with COL15A1 expression, clinical phenotypes such as the age of the patient and tumor stage, were chosen. p < 0.05 was taken to be statistically significant when utilizing the R packages “limma” and “ggplot2” to perform clinical phenotypic correlation analysis.

### Correlation of COL15A1 expression levels with TMB, MSI and MMR

TMB (tumor mutation burden) is an important component in determining the number of mutations in tumor cells [10], and MSI (microsatellite instability) is a prognostic indicator linked to the lack of MMR (mismatch repair) [11]. To obtain the TMB score, a Perl script was used to calculate and rectify the data. MSI scores were also calculated using somatic mutation data obtained from the USCS. The link between COL15A1 expression and MSI and TMB was then investigated making use of Spearman's rank correlation coefficient. Using the R packages, the analytic findings were presented as a heat map and a radar chart. Down-regulation or functional abnormalities of MMR genes may result in a higher frequency of somatic mutations and an increased risk of cancer [12]. The expression levels of MMR genes, PMS2, EPCAM, MLH1, and MSH2 in different cancers were evaluated by the expression profile data from UCSC, and the correlation of expression levels between MMR gene and COL15A1 were analyzed using the same expression profile data. Findings are given as a heatmap, created employing the R-packages, “RColorBrewer.” and “reshape2”.

### The relationship between COL15A1 expression level and immunity

ESTIMATE (Estimation of Stromal and Immune Cells in Malignant Tumor Tissues Using Expression Data) was implemented to determine the degree of stromal or immune cell infiltration [13, 14]. The ESTIMATE method was employed for generating the stromal and immune scores for individual tumor specimens, and the R software packages “limma” and “estimate” were utilized to assess the connection between the expression of COL15A1 and the two scores based on the degree of immune infiltration. We assessed the immune cell expression levels and investigated the correlation between immune cells for all samples in 33 types of malignancies making use of this method. In addition, the relative scores of 26 immune cells in 33 tumors were calculated using the CIBERSORT algorithm. The R packages “ggplot2” and “ggpubr” were used to assess the relationships between the levels of COL15A1 and individual levels of immune cell infiltration in the tumor (P 0.05 was considered significant). Then, using TIMER 2.0 (http://timer.cistrome.org/), we looked at the closeness between COL15A1 expression levels and cancer-associated fibroblasts (CAFs) in 33 different tumor types.

The R-package “limma” was also employed for carrying out the co-expression assessment of COL15A1 and immune-related genes, such as chemokine receptor proteins and the genes encoding major histocompatibility complex (MHC). The results are shown as a heatmap, which was generated by making use of R packages “reshape2” and “RColorBreyer.”

### The relationship between COL15A1 and CSCs

Cancer stem cells (CSCs) serve an important role in the formation and growth of cancer which may be considered as a risk factor for the prognosis of tumor patients [15]. Stem cell expression profile data were extracted from UCSC (https://xenabrowser.net/datapages/), and we analyzed the relationship between COL15A1 CSCs from the level of RNA expression and DNA methylation by the data from UCSC. Visualizing the analysis results as heatmap, using the R-packages “corrplot” to obtain them.

### Drug sensitivity analysis

The CellMiner (https://discover.nci.nih.gov/cellminer/home.do) was used for obtaining drug sensitivity data and gene expression profiles. The R packages “imput” and “limma” were employed for drug sensitivity assessment, and the R packages “ggplot2” and “ggpubr” were utilized for illustrating the association between drug sensitivity and COL15A1 expression level.

### GSEA enrichment analysis

We used Gene Set Enrichment Analysis (GSEA) for investigating the biological activities of COL15A1 in cancers, using data from the Gene Ontology (GO) and Kyoto Encyclopedia of Genes and Genomes (KEGG) gene sets available on the official GSEA website (https://www.gsea-msigdb.org/gsea/downloads.jsp). The R packages “clusterProfiler,” “enrichplot”, “limma,” “org.Hs.eg.db,” and were employed for performing functional analysis.

### Analysis of COL15A1 gene mutation

SNPs (Single Nucleotide Polymorphisms) are DNA sequence polymorphisms arising at the genomic level from the difference of a single nucleotide, and they may raise the risk of tumor growth. The data of SNPs were downloaded from TCGA and we employed perl and R scripts to process the outcomes. The R packages such as “GenVisR” and “reshape2” were used to present the analysis results. In addition, we explored COL15A1 gene variants and the prognosis of 33 tumors using the cBioportal tool (http://cbioportal.org).

### Analysis of genes associated with COL15A1

In an attempt to further probe into the molecular mechanism of the COL15A1 gene in tumorigenesis, 50 COL15A1 proteins were screened with experimental support from the STRING tool (https://string-db.org/). Subsequently, we selected the 100 genes, majorly associated with COL15A1 in GEPIA2, and plotted the scatter plot. Later, we obtained a heat map of the correlation between COL15A1 and these genes by TIMER 2.0. To observe the genes further, the veen website (http://bioinformatics.psb.ugent.be/webtools/Venn/) was employed to take the intersection of the top100 genes and the 50 genes interacting with the protein and screened for two genes, precisely SLIT3 and HSPG2. Thereafter, we evaluated the biological functions of these genes using K-M survival analysis.

### Cell culture

Human normal liver cells (L02) and hepatocellular carcinoma cells (HepG2, LM3 and Huh7) were acquired from Procell Life Science & Technology Co, Ltd (Wuhan, China). These cell lines were cultured in DMEM supplemented with 10% FBS, 1% penicillin and 1% streptomycin, and the cells were grown at 37 ℃ with 5% CO_2_.

### Quantitative real-time PCR (qRT-PCR)

Total RNA was extracted from cells with TRIzol reagent (Life Technologies, USA) according to the manufacturer's instructions.NanoDrop 2000 was used to determine the concentration of RNA. Subsequently, PrimeScript RT Master Mix (Takara, Japan) was used to synthesise cDNA. cDNA was synthesised using TB Green Premix Ex Taq (Takara, Japan) on an ABI PRISM 7900 Sequence Detection System (Applied Biosystems, Carlsbad, USA) to complete qRT-PCR.

### COL15A1 expression construct and cell transfection

The full-length cDNA of human Col15a1 (NM_001855) was inserted into the BamHI/AgeI sites of p-GV492, purchased from JiKai Company (Shanghai, China). An empty p-GV492 was used as a control. The cells were transfected with the lentivirus at a MOI of 5 × 10^8^ TU/mL in the presence of 5 µg/mL polybrene (HanHeng, Shanghai, China), and successfully transfected cells were selected using 1 µg/mL puromycin (HanHeng, Shanghai, China).

### Western blot analysis

After completion of cell transfection, cells were lysed with RIPA buffer containing a phosphatase inhibitor cocktail. Proteins were loaded and electrophoretically separated on SDS polyacrylamide gel electrophoresis (SDS-PAGE) and then transferred to nitrocellulose membranes. Primary antibodies were added to bind the corresponding proteins overnight at 4 °C. Subsequently, the membrane was incubated with HRP-conjugated secondary antibody (Absin, Shanghai, China) for 1 h at room temperature and exposed to ECL reagent (NCM Biotech, Suzhou, China).

### CCK8 assay

HepG2, LM3 and cell lines overexpressing COL15A1 were inoculated into 96-well plates in equal numbers. 10 µL of CCK solution was added to the cells at a ratio of CCK-8 solution: medium = 10:100. After 2 h of incubation, optical density (OD) values were measured at 450 nm using a multifunctional microplate reader.

### Wound healing assay

Wound healing assays were used to observe the migratory efficiency of the cells, and cells overexpressing HepG2 and LM3 reached 100% confluence with a 10 µL pipette to form a wound in the centre of the cell monomer. Subsequently, the culture was continued in the incubator and the wound area was calculated at the same intervals using Image J software.

### Cell invasion assay

Cell invasion efficiency was assessed using the Transwell assay. Transfected cells were collected, resuspended in serum-free medium, and approximately 2 × 10^4^ cells containing 300 µL of serum-free DMEM were placed into the upper chamber, while 500 µL of DMEM supplemented with 10% FBS was added to the lower chamber. 48 h later, cells adhering to the lower membrane were then fixed with 4% paraformaldehyde and stained with crystal violet. The surface of the upper membrane was wiped with a cotton swab to remove residual cells. The cells were then photographed and analysed using a light microscope.

### Statistical analysis

Statistical analyses were performed using two-tailed unpaired analyses. GraphPad Prism software was used for statistical analysis. p < 0.05 was regarded statistically significant. R software was used to perform all data analysis (version 4.1.0).

## Results

### Differential expression of COL15A1

**Fig. 1:**
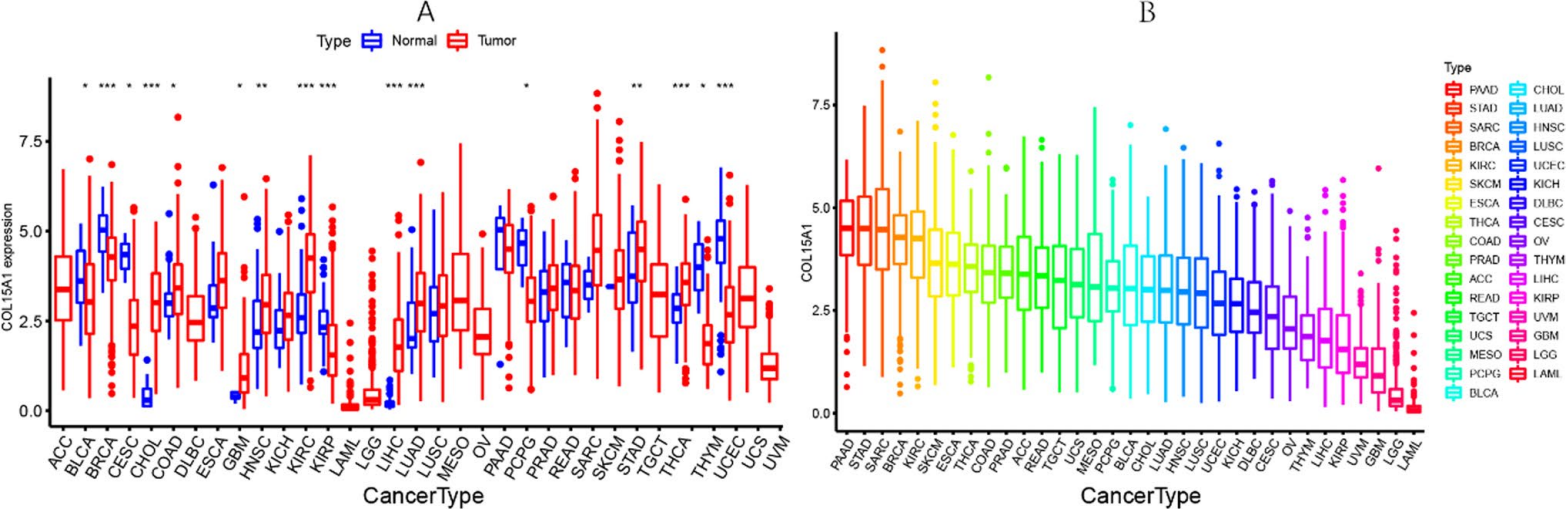
Differential expression of COL15A1. **A** Comparison of COL15A1 expression between tumor and normal samples. *P < 0.05, **P < 0.01, ***P < 0.001. **B** COL15A1 expression in 33 kinds of cancer

Based on data from the UCSC, we evaluated the expression levels of COL15A1 in 33 cancers and normal tissues (Fig. 1A). Except for the cancers for which no normal tissue data is available, COL15A1 expression in tumors and normal tissues differs considerably in all but one of the 19 tumors studied. COL15A1 was observed to have an elevated expression in several cancers, including cholangiocarcinoma (CHOL), glioblastoma multiforme (GBM), colon adenocarcinoma (COAD), thyroid carcinoma (THCA), head and neck squamous cell carcinoma (HNSC), esophageal carcinoma (ESCA), lung adenocarcinoma (LUAD), kidney renal clear cell carcinoma (KIRC), liver hepatocellular carcinoma (LIHC) kidney chromophobe (KICH), stomach adenocarcinoma (STAD), Sarcoma (SARC). On the other hand, COL15A1 levels were revealed to be decreased in some tumors when compared to normal tissues, including bladder urothelial carcinoma (BLCA), breast invasive carcinoma (BRCA), kidney renal papillary cell carcinoma (KIRP), thymoma (THYM), cervical squamous cell carcinoma, and endocervical adenocarcinoma (CESC), Uterine Corpus endometrial carcinoma (UCEC), and pancreatic adenocarcinoma (PAAD). The greatest significant variations in COL15A1 expression between normal and tumor tissues were found in the KIRC and THYM groups. However, the variation in COL15A1 expression between cancers and normal tissues was not statistically significant in some malignancies, such as prostate adenocarcinoma (PRAD) and rectum adenocarcinoma (READ).

In addition, the expression levels of COL15A1 was assessed in several carcinomas and were classified from high to low in terms of their significance (Fig. 1B). All malignancies expressed COL15A1, and the highest levels of COL15A1 expression were found in PAAD while the lowest levels were found in acute myeloid leukemia (LAML).

### Immunohistochemistry (IHC) staining

**Fig. 2:**
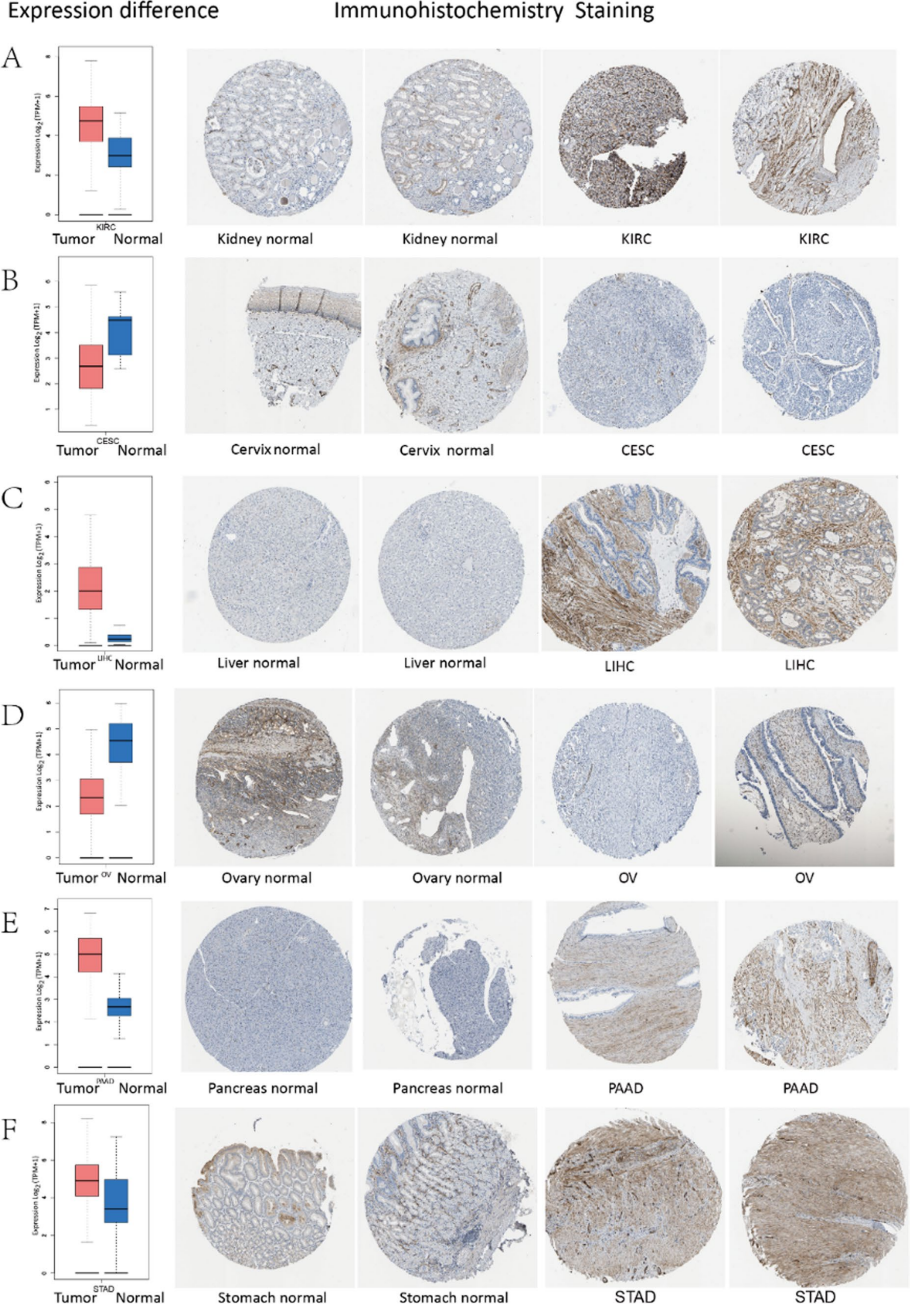
Expression of COL15A1 in normal and tumor tissues (left) and comparison of immunohistochemical photographs of tumor tissues (right) and normal (middle). The expression of COL15A1 protein was substantially lower in Endocervical adenocarcinoma (CESC) and Ovarian serous cystadenocarcinoma (OV) tissues than normal tissues

Our research team evaluated IHC results from the HPA database and correlated them to gene expression data from the TCGA in order to determine COL15A1 protein expression levels at the protein level. An illustration of the findings is shown in Figs. 2A–F. IHC staining of normal stomach, kidney, liver, and pancreatic tissues revealed modest COL15A1 staining, whereas tumor tissues revealed significant staining. Normal cervix and ovary tissues, on the other hand, showed significant COL15A1 staining, whereas tumor tissues showed only moderate staining (Fig. 2D).

### Analysis of COL15A1 in tumor prognosis

**Fig. 3:**
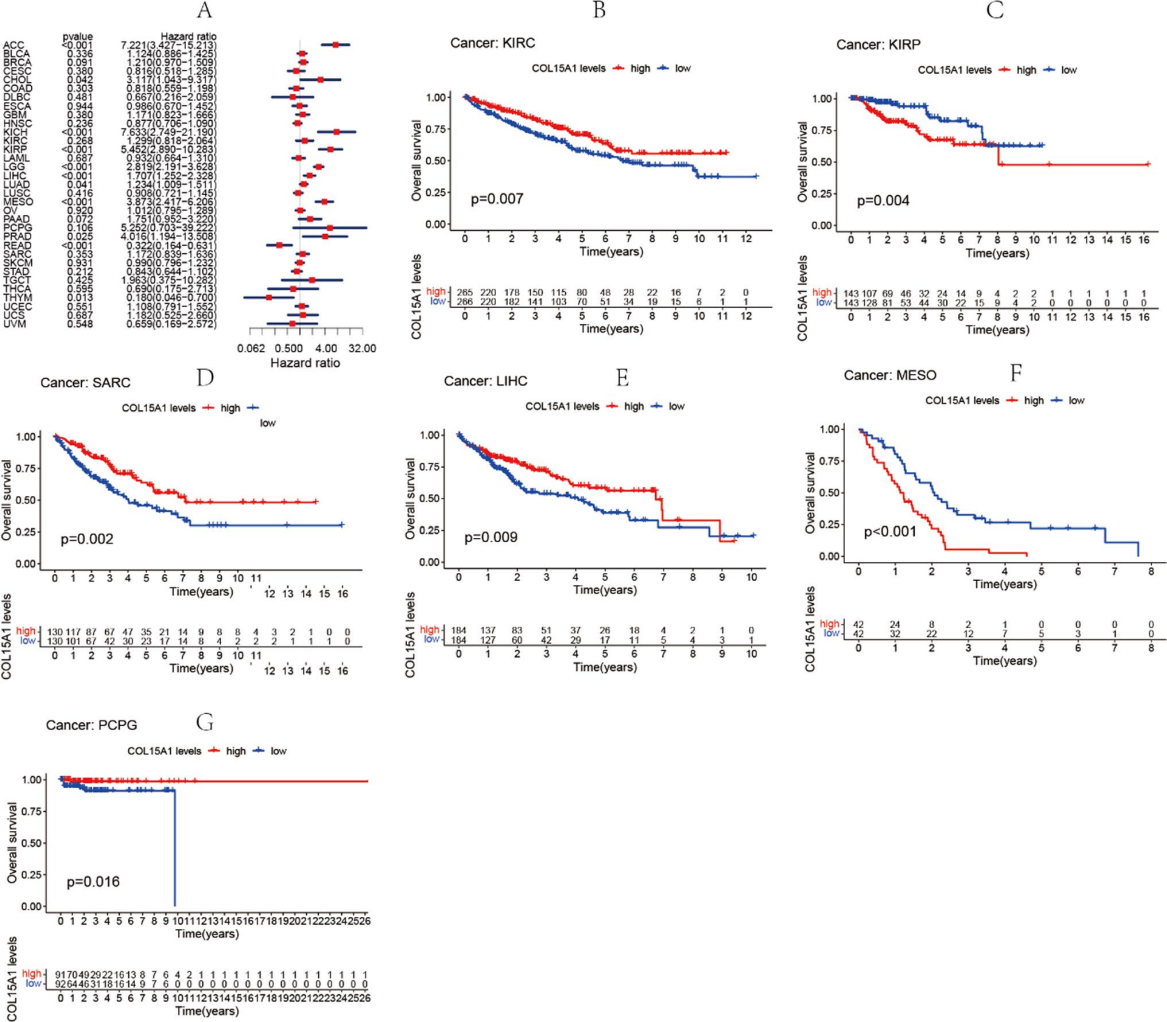
Association between overall survival (OS) time in days and COL15A1 expression. **A** Forest plot of OS correlations in 33 different tumors. **B**–**G** Kaplan–Meier assessment of the relationship between OS and COL15A1 expression

To investigate COL15A1 expression levels are related to tumor prognosis, a survival correlation analysis was performed for each tumor using four metrics; precisely, OS, DSS, DFI, and PFI. COL15A1 expression levels were correlated with OS in brain lower-grade glioma (LGG) (p < 0.001), LIHC (p < 0.001), adrenocortical carcinoma (ACC) (p < 0.001), CHOL (p = 0.042), KICH (p < 0.001), KIRP (p < 0.001), LUAD (p = 0.041), mesothelioma (MESO) (p < 0.001), PRAD (p = 0.025), READ (p < 0.001), THYM (p = 0.013) as shown by cox proportional hazard model analysis in Fig. 3A. Moreover, in ACC, CHOL, KICH, KIRP, LIHC, MESO, PRAD, COL15A1 was a high-risk gene, however, it was a low-risk gene in READ and THYM. We could confirm the conclusion in KM Survival that low COL15A1 expression demonstrated an association with shorter survival times in KIRC (Fig. 3B, p = 0.007), LIHC (Fig. 3E, p = 0.009), SARC (Fig. 3D, p = 0.002), PCPG (Fig. 3G, p = 0.016). However, low levels of COL15A1 had better OS in KIRP (Fig. 3C, p = 0.004), MESO (Fig. 3F, p < 0.001).

**Fig. 4:**
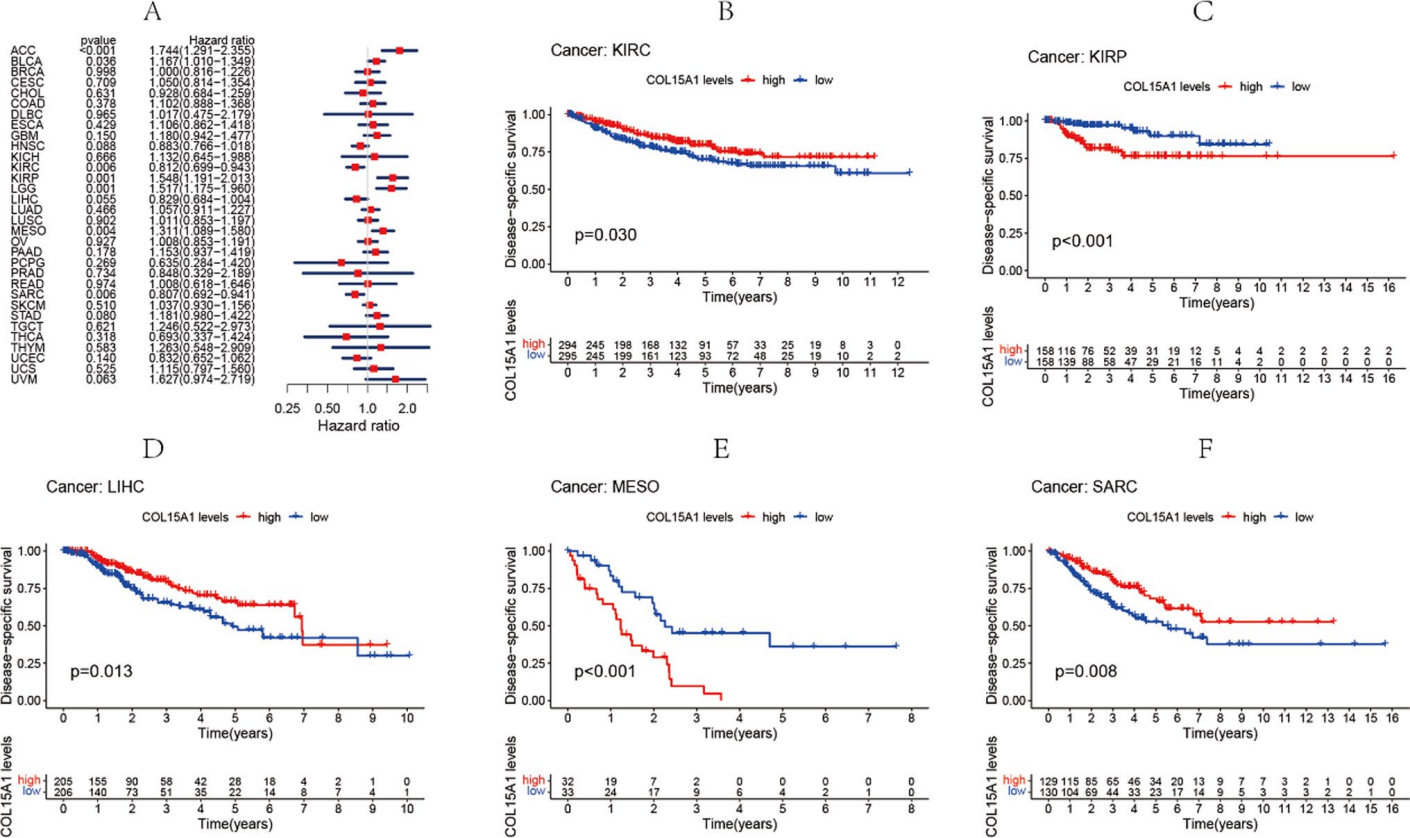
Association between the disease-specific survival (DSS) and expression of COL15A1. **A** Forest plot of DSS associations in 33 different tumors. **B**–**F** Kaplan–Meier assessment of the association between DSS and COL15A1 expression

Furthermore, the COX analysis of DSS data divulged that increased expression of COL15A1 was relevant to an unfavorable prognosis in individuals suffering from ACC (p < 0.001), BLCA (p < 0.036), KIRP (p < 0.001), and LGG (p < 0.001), among other diseases (Fig. 4A). In SARC, the expression of COL15A1 had an inverse connection with prognosis (p = 0.006), although the expression of COL15A2 did not. As revealed by the outcome of the KM survival analysis, increased expression of COL15A1 was relevant to a bad prognosis in both KIRP and MESO (Fig. 4C, E). The high expression of COL15A1 in KIRC, LIHC, and SARC, on the other hand, was associated with a prolonged survival time (Fig. 4B, D, F).

**Fig. 5:**
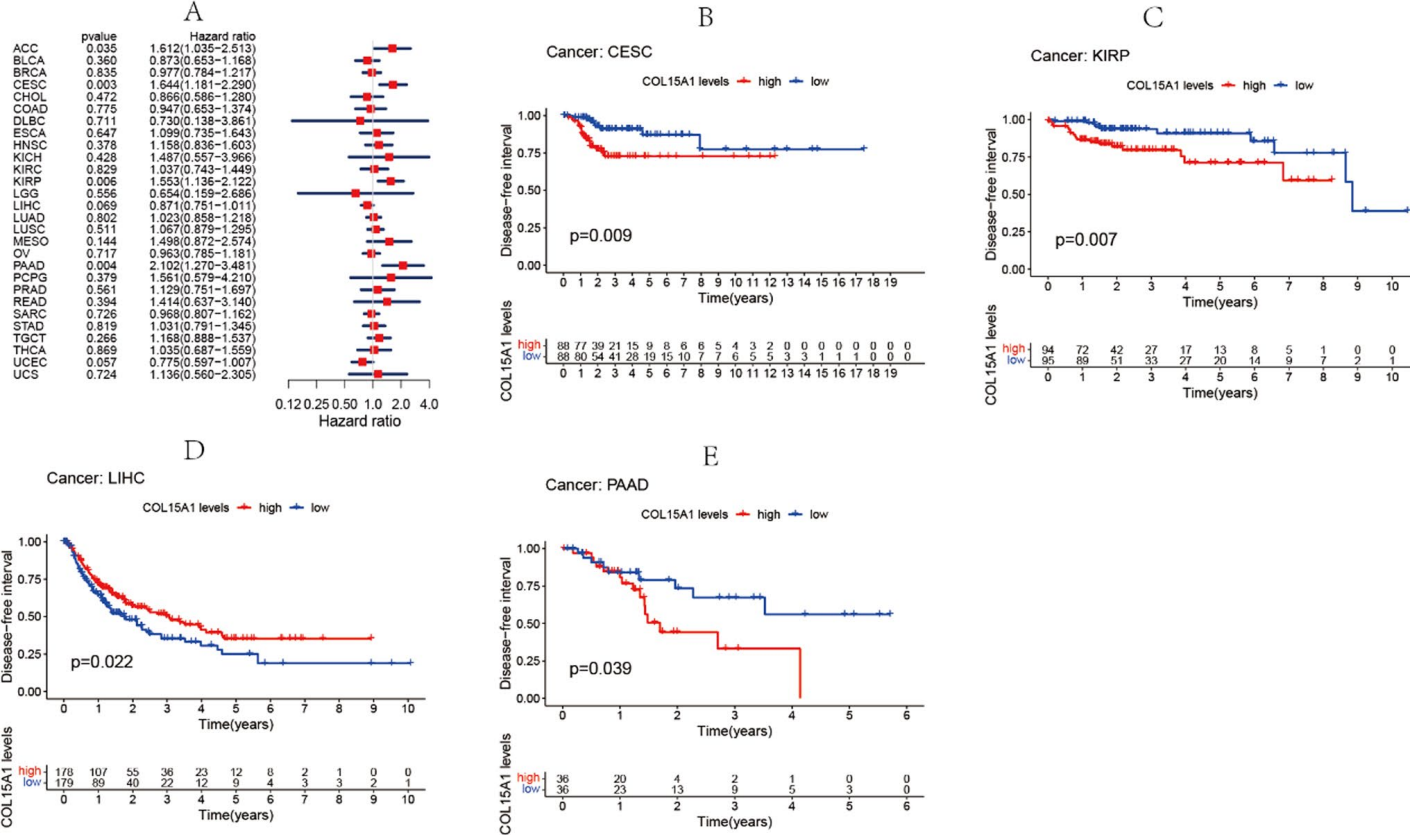
Association between COL15A1 expression and disease-free interval (DFI). **A** Forest plot of DFI associations in 33 different tumors. **B**–**E** Kaplan–Meier assessment of the association between DFI and COL15A1 expression

**Fig. 6:**
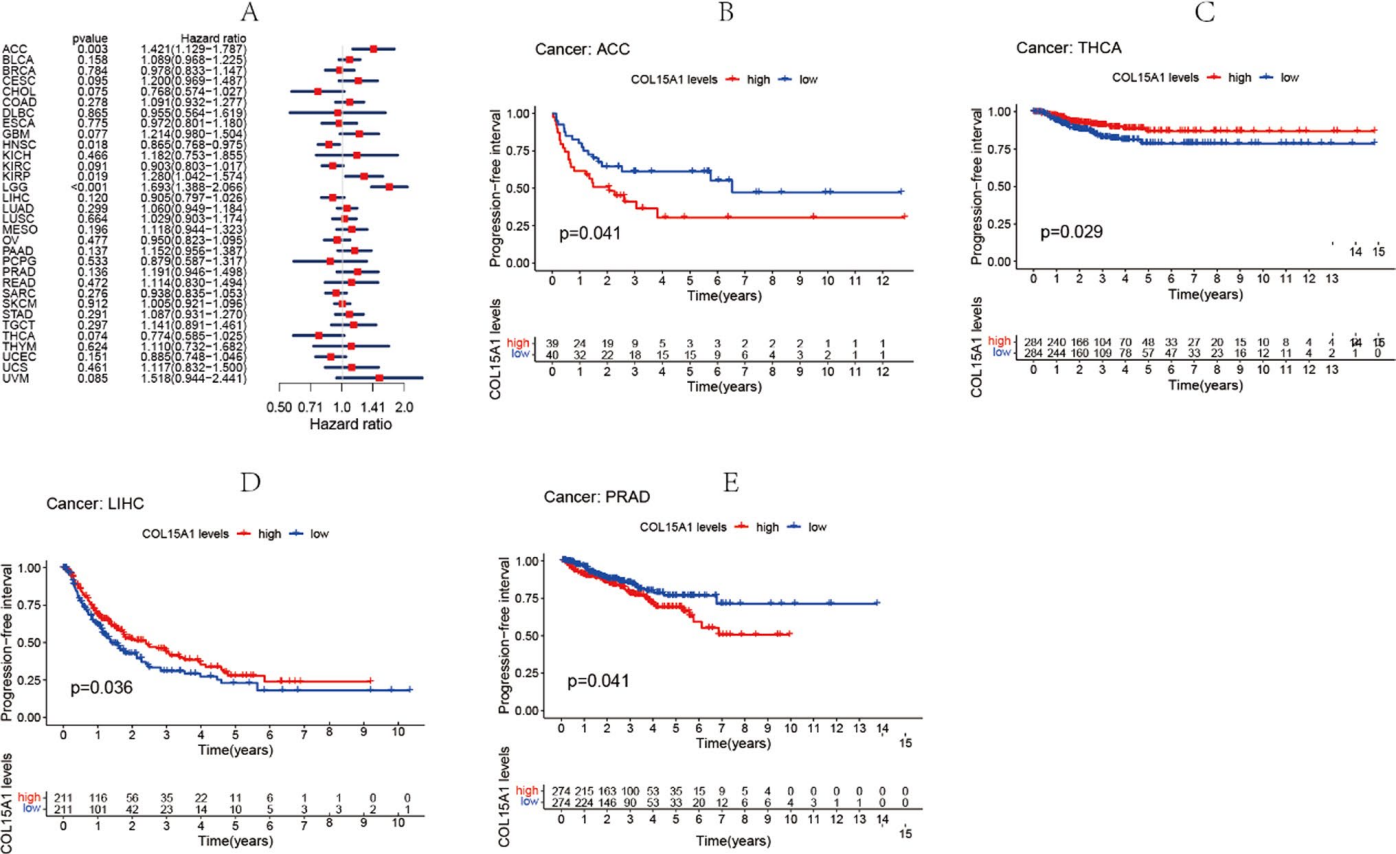
Association between the progression-free interval (PFI) and the expression of COL15A1. **A** Forest plot of PFI association with the expression of COL15A1 in 33 different tumors. **B**–**E** Kaplan–Meier assessment of the association between the PFI and expression of COL15A1

Similarly, an identical methodology is employed for analyzing the data of DFI and PFI. In the diverse sorts of cancers, detection of the closeness between COL15A1 expression and DFI was carried out (Fig. 5A), including ACC (p = 0.035), CESC (p = 0.003), KIRP (p = 0.006), PAAD (p = 0.004). On the other hand, significant relationships were identified in CESC (p = 0.009), KIRP (p = 0.007), LIHC (p = 0.022), PAAD (p = 0.039) by survival curve (Fig. 5B–E). On the relationship between PFI and expression of COL15A1, forest plots revealed correlations between considerable expression and poor PFI in ACC (p = 0.003), HNSC (p = 0.018), KIRP (p = 0.019), LGG (p < 0.001) (Fig. 6A). Nevertheless, no correlation existed between COL15A1 low-expression and PFI in any kind of cancer. For KM survival analysis (Fig. 6B–E), the low expression of COL15A1 represents a poor prognosis in THCA and LIHC, while in ACC and PRAD, the low expression of COL15A1 means a longer survival time.

### Correlation analysis between COL15A1 expression and clinical phenotype

**Fig. 7:**
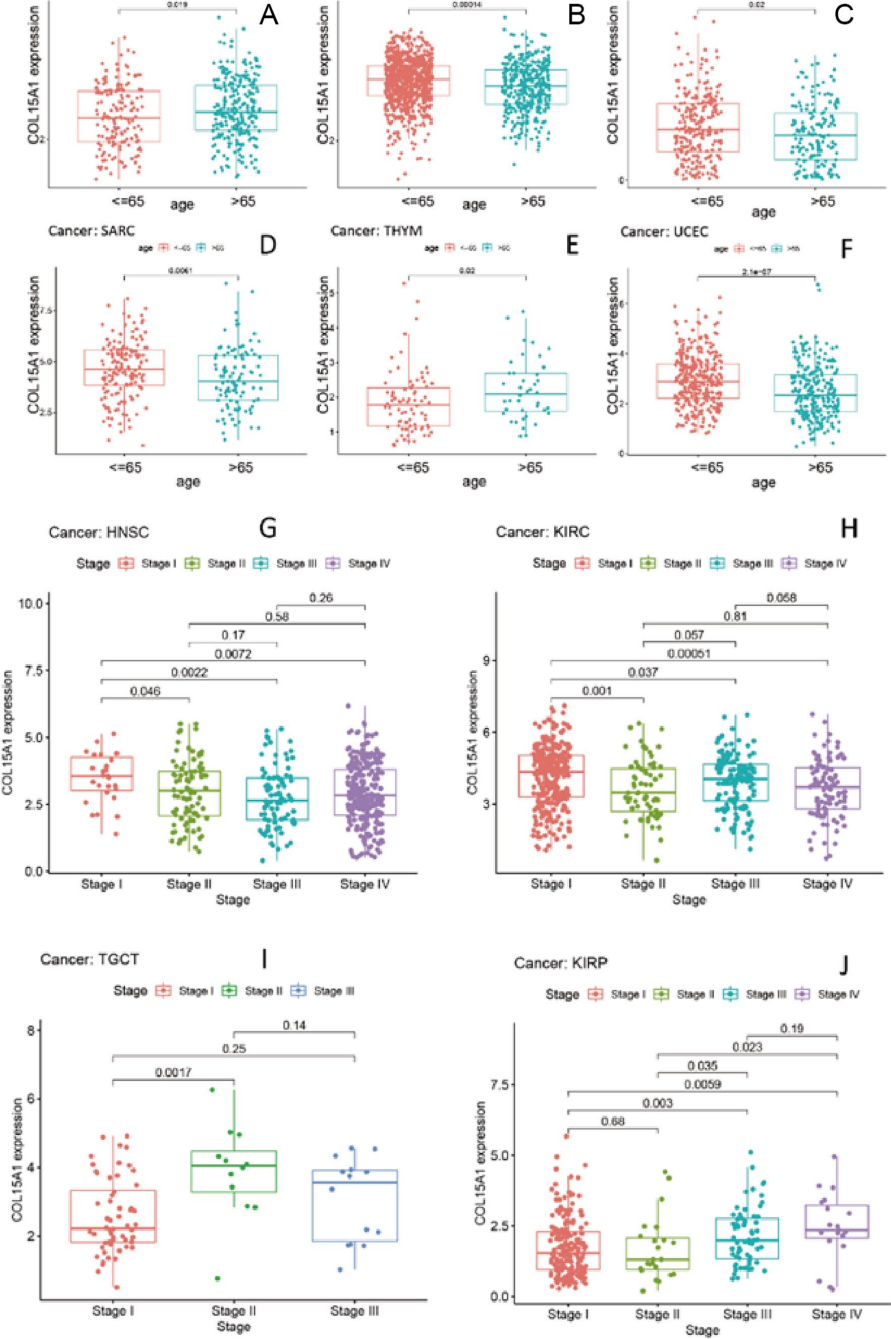
Correlation between COL15A1 expression tumor stage and age in multiple tumors. **A**–**F** relationship between COL15A1 expression and age in tumor tissues. **G**–**J** relationship between COL15A1 expression in tumor tissues and tumor stage

We looked into the association between the levels of gene expression and the age of the patient (Fig. 7A–F) and the stage of the tumor (Fig. 7G–J). With respect to the tumor stage, a statistically significant relationship was detected between the tumor stage and four types of cancer, including HNSC, KIRC, KIRP, and testicular germ cell tumors (TGCT). Interestingly, significant variations in gene expression are found mostly between stage I and stage II cancers (Fig. 7G-I), with some differences being found between stage I and stage III tumors (Fig. 7G–J). However, in the vast majority of cancers, the closeness between gene expression levels and tumor stage is meaningless or non-existent. UCEC (p = 2.1e−0.7), BRCA (p = 0.00014), SARC (p = 0.0061), BLCA (p = 0.019), LIHC (p = 0.02), and THYM (p = 0.02) were the six cancers studied that were most linked with COL15A1 expression levels to present the findings. All of these findings imply that there is a statistically significant relationship between COL15A1 expression levels and tumor stage, as well as patient age, in specific types of tumors.

### Correlation of COL15A1 expression levels with TMB, MSI and MMR

**Fig. 8:**
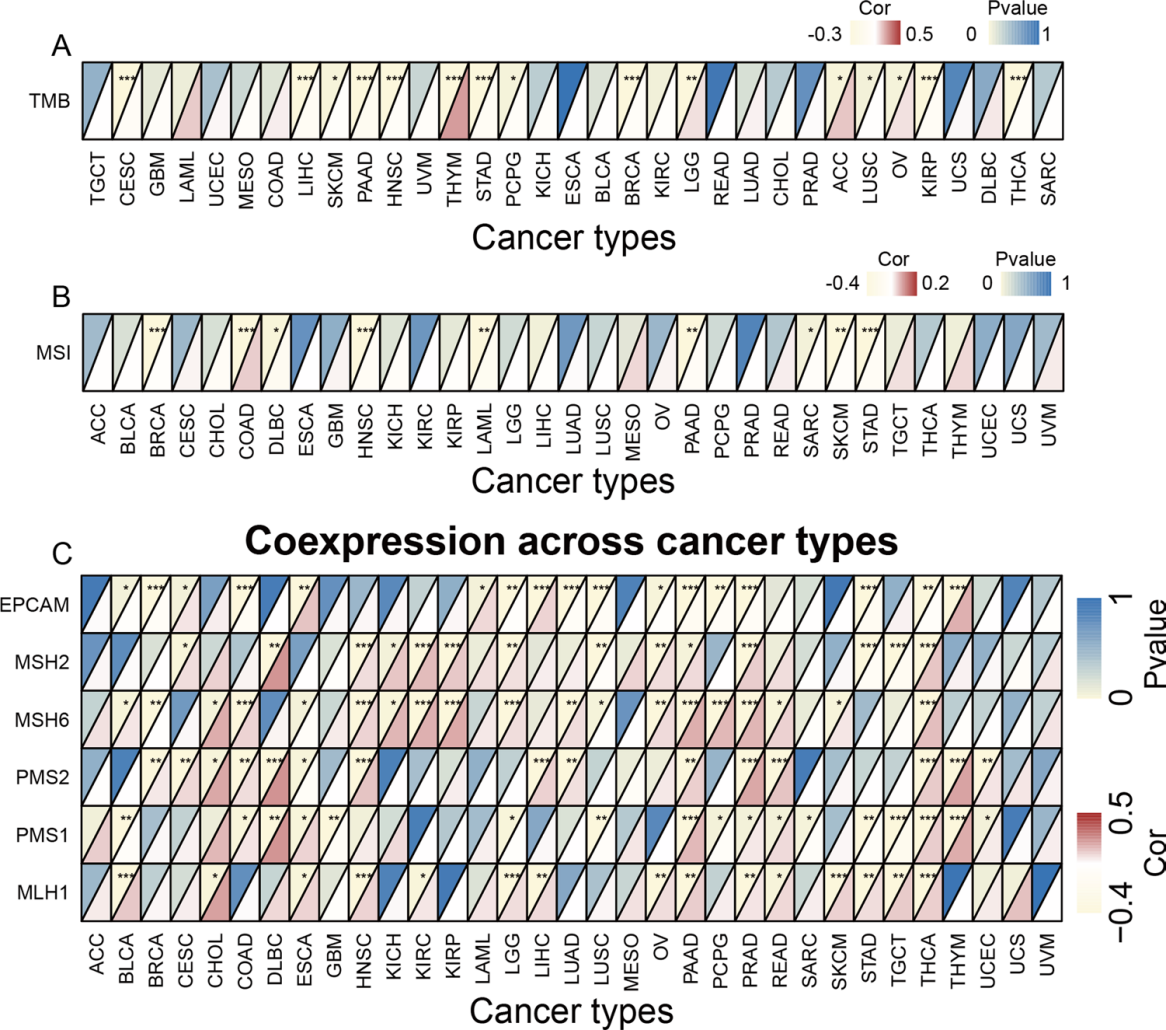
Associations between tumor microsatellite instability (MSI), mutational burden (TMB), and mismatch repair (MMR) and COL15A1 expression. **A** Heatmap showing the association between TMB and COL15A1. The top left triangle, for each pair represents the P-value, and the correlation coefficient is represented by the bottom-right triangle; ***p < 0.001, **p < 0.01, and *p < 0.05. **B** Heatmap showing the association between MSI and COL15A1. The top left triangle, for each pair represents the P-value, and the correlation coefficient is represented by the bottom-right triangle; ***p < 0.001, **p < 0.01, and *p < 0.05. **C** Heatmap showing the association between MMR genes and COL15A1 expression. The top left triangle, for each pair represents the P-value, and the correlation coefficient is represented by the bottom-right triangle; ***p < 0.001, **p < 0.01, and *p < 0.05

Following that, we looked at the relation among COL15A1 expression level and TMB, MSI, and MMR, which were all associated with the susceptibility to immune checkpoint inhibitors (IKIs). So we looked into the relationship between MMR genes (such as MLH1, MSH2, PMS1, PMS2, EPCAM, and PMS6) and TREM2 gene levels (as well as the relationship between MMR genes and TREM2 gene levels). The findings depicted that the expression of COL15A1 has an association with the tumor microenvironment (TME) in 15 types of cancers (Fig. 8A), including breast cancer, liver cancer, and pancreatic carcinoma. Another 9 types of malignancies, including breast cancer and colorectal cancer, have high levels of COL15A1 expression that are associated with high MSI levels (Fig. 8B). Aside from that, we looked at the relation between COL15A1 expression levels and the level of MMR gene expression (Fig. 8C). A statistically significant positive association is available between the MMR gene expression level and COL15A1 expression levels in various cancers, such as CHOL and Lymphoid Neoplasm Diffuse Large B-cell Lymphoma (DLBC). Another tumor type, such as GBM and LUSC, had an opposing association of gene expression levels, which was discovered by correlation of gene expression levels.

**Fig. 9:**
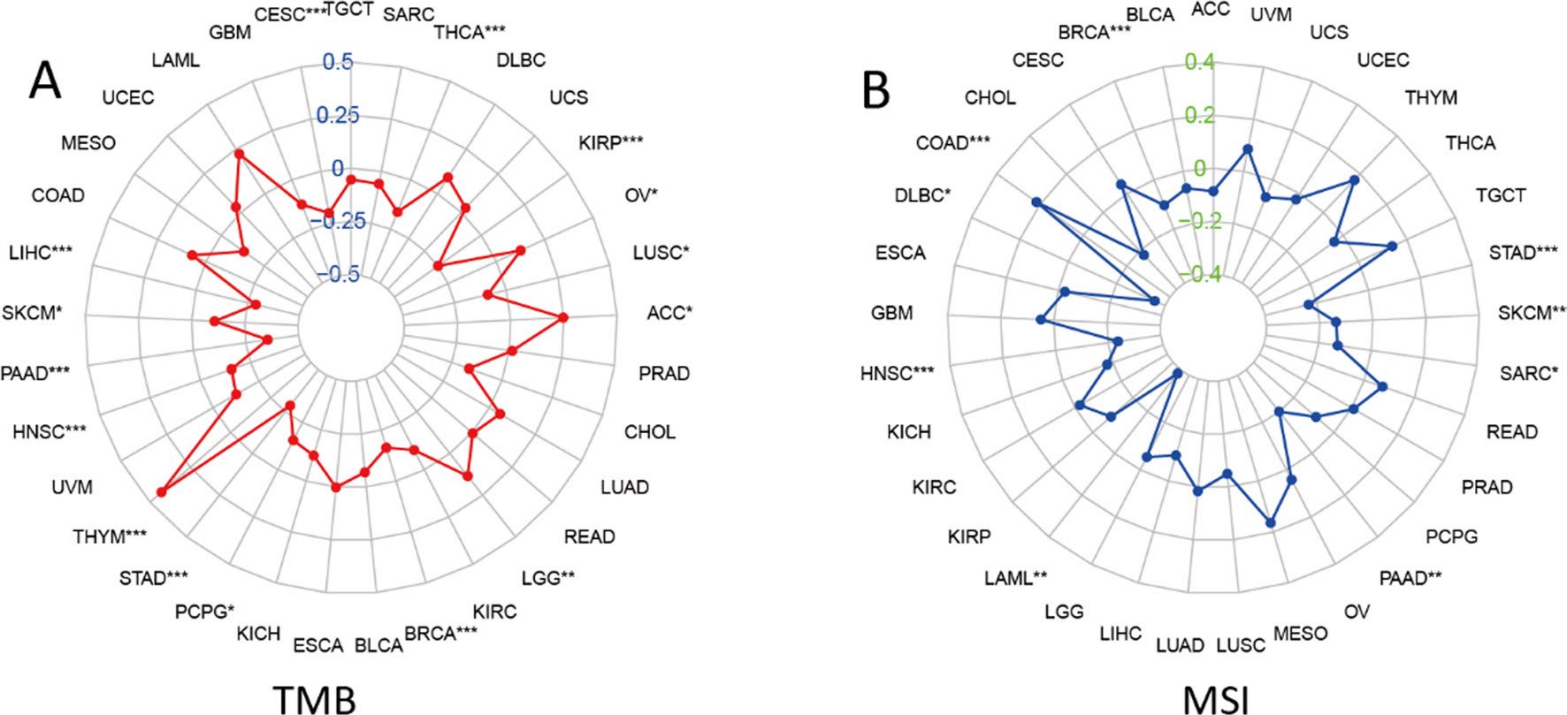
Association between **A** tumor mutational load (TMB), **B** microsatellite instability (MSI) and COL15A1 expression and the number on the circle indicates the correlation coefficient and * indicates the p-value, ***p < 0.001, **p < 0.01, and *p < 0.05

Radar plots were also used to highlight the relation among COL15A1 expression levels, TMB, and MSI. In ACC and THYM, COL15A1 expression levels were considerably positively connected with TMB, but they were significantly negatively correlated in PAAD and KIRP (Fig. 9A). In DLBC and LAML, COL15A1 expression levels were significantly positively linked with MSI, however, this association was reversed in COAD (Fig. 9B). Importantly, all of these findings corroborated Fig. 8’s conclusion, indicating that our methodology was correct.

### COL15A1 and the tumor microenvironment (TME)

**Fig. 10:**
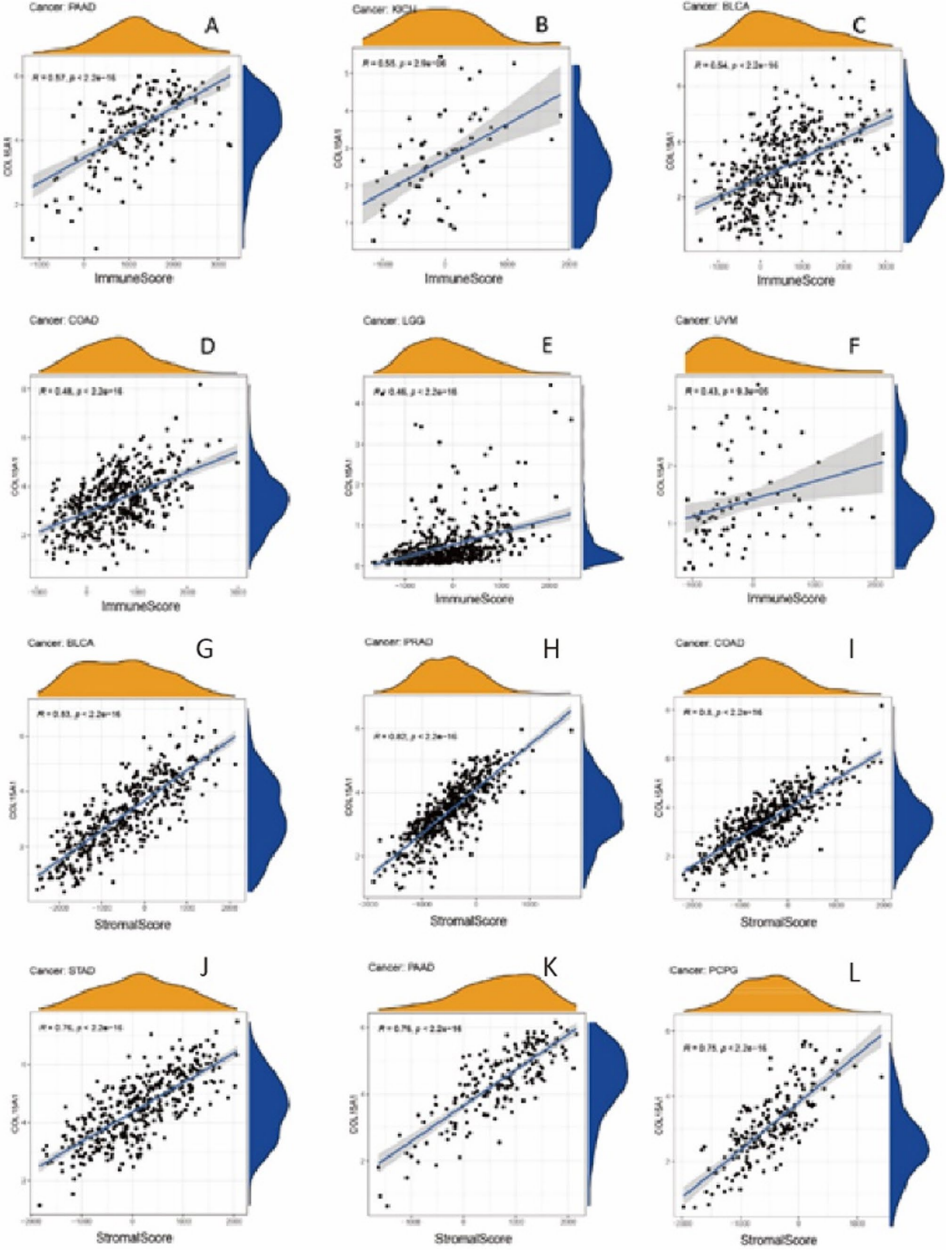
Six tumors with the highest correlation coefficient between COL15A1 expression and tumor microenvironment. **A**–**F** represents the correlation between COL15A1 and immune scores; **G**–**L** represents the correlation between COL15A1 and stromal scores

TME is linked to the occurrence and progression of malignancies [16]. Consequently, we explored the link between TME and COL15A1 expression levels across cancer types. The ESTIMATE algorithm was employed for estimating the stromal and immune cell scores for 33 tumors, as well as the association between the two scores and gene expression levels (Fig. 10). In PAAD, KICH, BLCA, COAD, LGG, and UVM, the achievements demonstrated that COL15A1 expression was significantly positively linked with immunological scores (Fig. 10A–F). Similarly, we obtained stromal scores in a pan-cancer assessment, and Fig. 10G–L shows the six tumors with the greatest coefficients of the correlation.

**Fig. 11:**
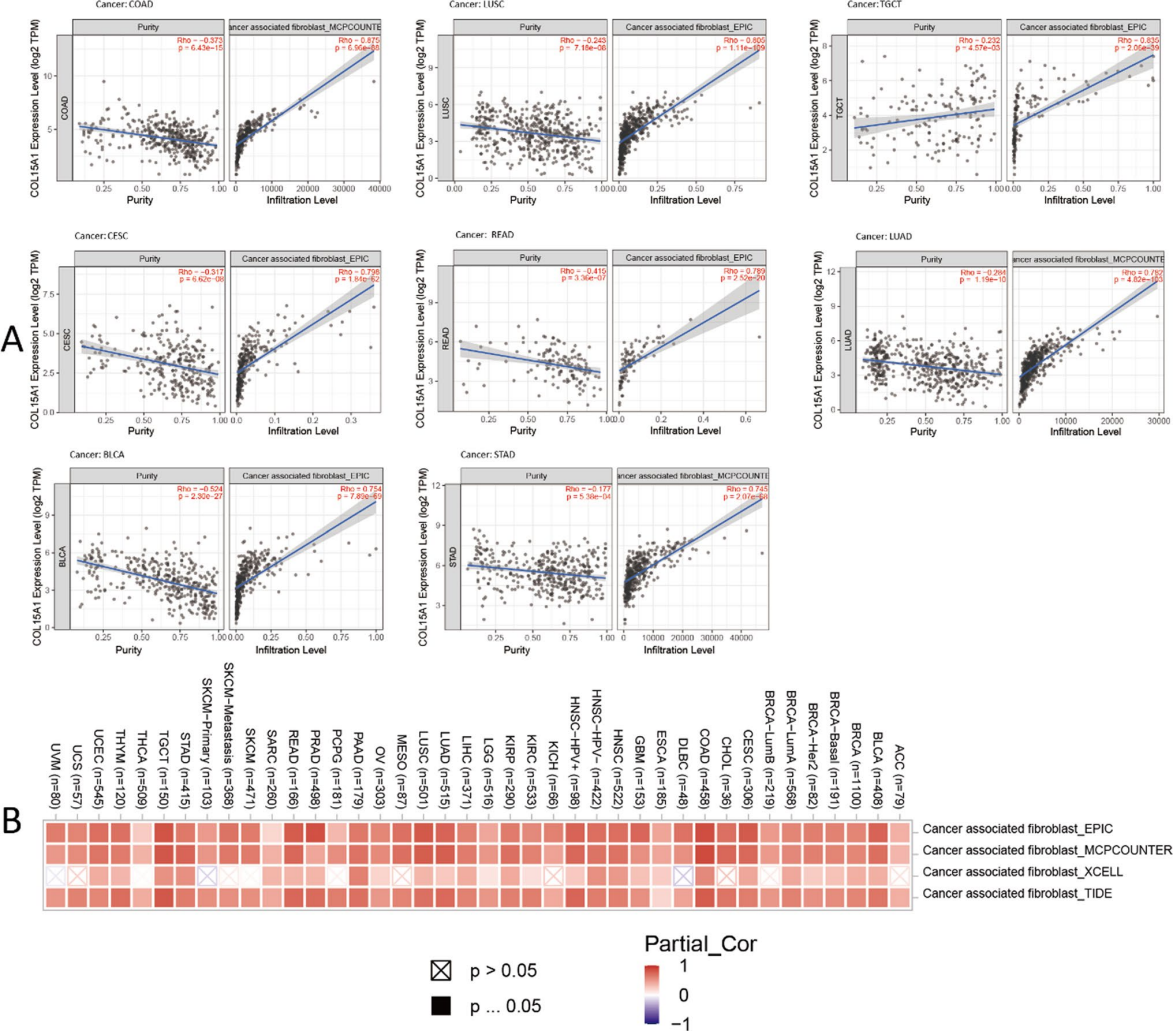
Correlation of COL15A1 expression in multiple tumors with cancer-associated fibroblasts (CAF). **A** Correlation of COL15A1 expression with tumor purity and CAF infiltration level in different tumors. **B** Multiple databases demonstrate the relationship between COL15A1 expression in different tumors and CAF infiltration

CAFs are critical in cancer progression when they are activated in the TME [17]. Here, the correlation between COL15A1 expression levels and CAFs is explored by TIMER 2.0, and the eight most relevant tumors are shown in Fig. 11A, including COAD, LUSC, TGCT, CESC, READ, LUAD, BLCA, STAD. Similarly, the heatmap (Fig. 11B) revealed that COL15A1 expression levels are strongly positively linked with CAFs in TGCT, COAD, CESC, and READ, confirming the previous research findings.

### COL15A1 and tumor immune cell infiltration

**Fig. 12:**
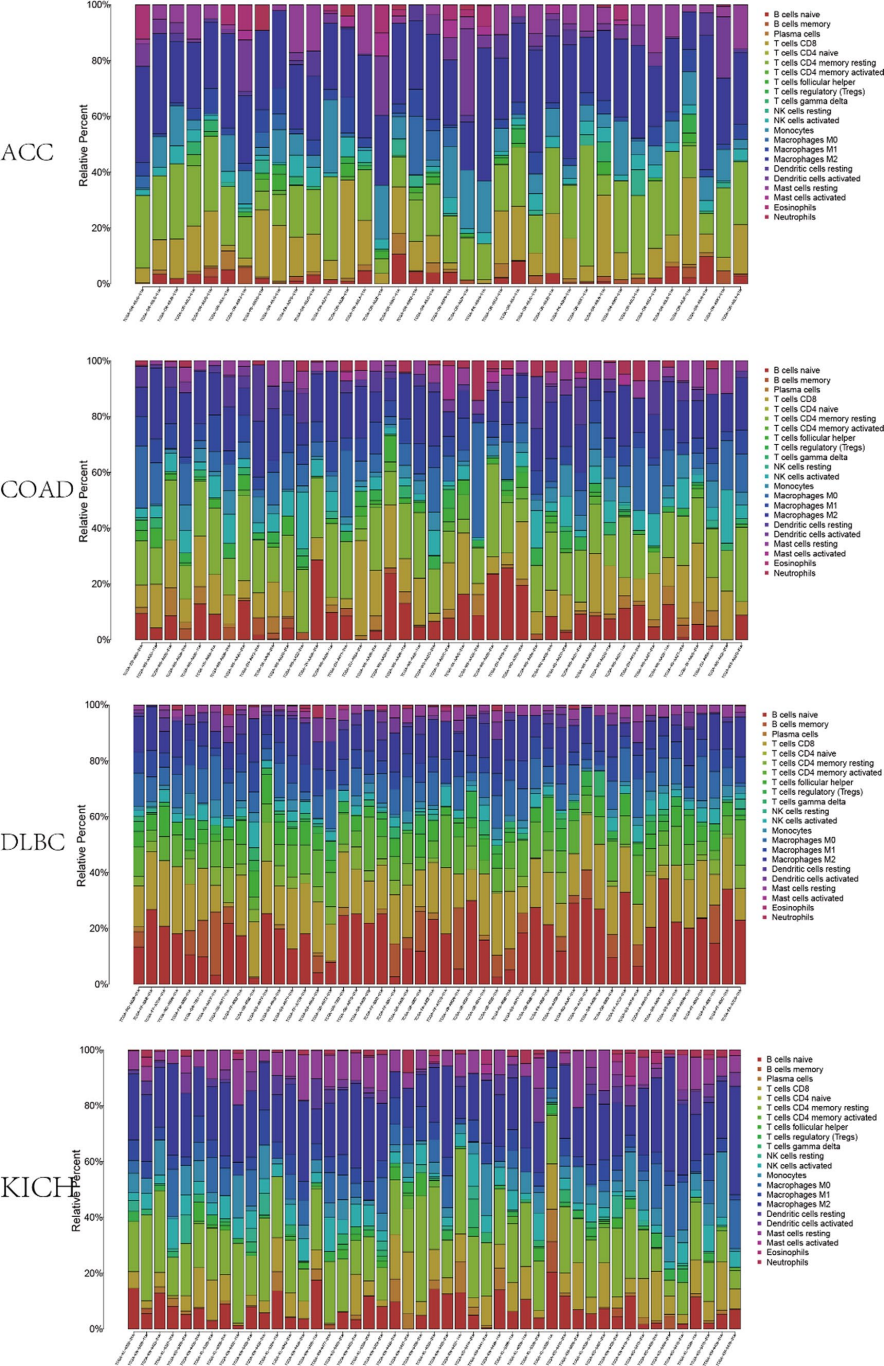
Expression of 22 types of immune cells in different tumors

**Fig. 13:**
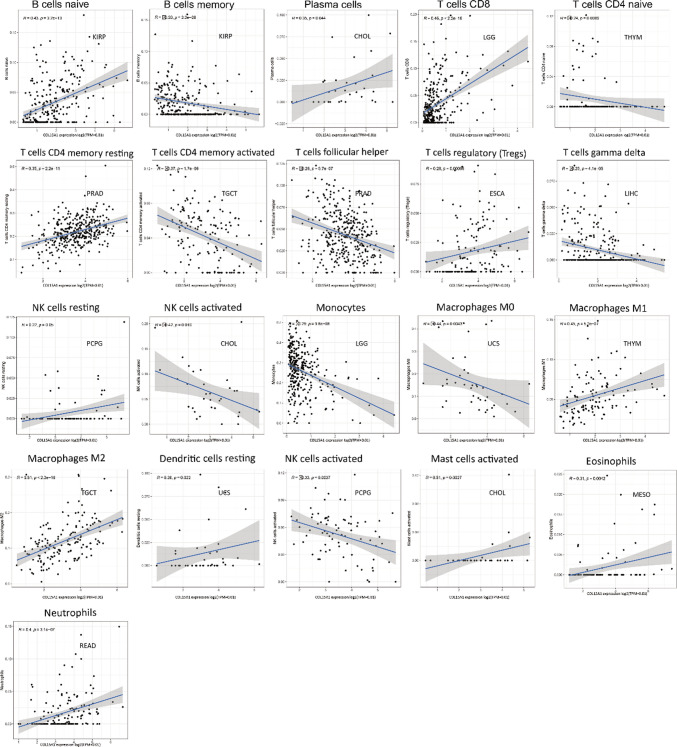
Correlation of COL15A1 expression with different immune cells in tumors, including NK cells, dendritic cells, B cells, T cells, neutrophils, etc.

We were able to identify the 22 different sorts of immune cells that were present in each tumor sample (Fig. 12). Then researchers looked at the link between COL15A1 and the amount of infiltration of 22 immune-related cells. Our findings revealed that there was a strong association between immune cell expression levels and COL15A1 expression levels in most cancers, including HNSC, KIRP, PRAD, BLCA, BRCA, ESCA, KIRC, COAD, and LGG (Table 1). The numbers of B memory cells and CD8 + T cells, also known as follicular helper T cells, were found to be adversely linked with COL15A1 expression. COL15A1 expression, on the other hand, was found to be positively linked with the number of naive B cells, CD4 + memory resting T cells, and resting mast cells. Furthermore, COL15A1 expression levels were shown to be positively linked with macrophage subtypes in the majority of cases. Furthermore, for each kind of immune cell in tumors, Fig. 13 indicated the strongest correlation coefficients between the COL15A1 expression and the degree of infiltration.

**Table 1:** Relationship between COL15A1 expression and immune cell infiltration in different tumors

Cancer type	HNSC (P/Cor)	KIRP (P/Cor)	PRAD (P/Cor)	BLCA (P/Cor)	BRCA (P/Cor)	ESCA (P/Cor)	KIRC (P/Cor)	COAD (P/Cor)	KIRC (P/Cor)	LGG (P/Cor)
B naïve cells	***/0.36	***/0.43	***/0.2	***/0.28	***/0.23	*/0.19	***/0.27		***/0.27	
B memory cells	***/− 0.16	***/− 0.33	**/-0.16	***/− 0.26	***/-0.1		***/− 0.16	***/− 0.21	***/− 0.16	
Plasma cells		**/0.17	***/− 0.2			*/0.22	*/-0.1		*/− 0.1	
CD 8^+^ T cells	*/− 0.094		***/− 0.31			**/− 0.22	***/− 0.16	*/− 0.1	***/− 0.16	***/0.46
CD 4^+^ naïve T cells				**/− 0.14						***/− 0.21
CD 4^+^ memory resting T cells	***/0.24		***/0.35		***/0.29	***/0.29	***/0.15	**/− 0.13	***/0.15	**/0.17
CD 4^+^ memory activated T cells	*/0.0015	***/0.25						**/− 0.13		**/0.17
Follicular helper T cells	***/− 0.17	*/0.15	***/− 0.26	***/− 0.25	***/− 0.19	*/− 0.2	***/− 0.22		***/− 0.22	
Regulatory T cells (Tregs)			**/0.14			***/0.28	***/− 0.17		***/− 0.17	*/0.12
Gamma delta T cells			*/0.12		***/0.11		**/− 0.13		**/− 0.13	*/0.12
Resting NK cells										
Activated NK cells	***/0.18	*/− 0.13	**/− 0.15	*/− 0.12	*/− 0.071	*/− 0.17	***/− 0.23		***/− 0.23	
Monocytes	***/0.21		**/0.14	**/0.14						***/− 0.29
M0 macrophages		**/− 0.17			**/− 0.0091			***/0.16		***/0.26
M1 macrophages		***/0.32	***/− 0.18					***/0.16		***/0.32
M2 macrophages	**/0.12	***/− 0.32		***/0.27	***/− 0.15		***/0.19	***/0.24	***/0.19	*/− 0.12
Resting dendritic cells	***/− 0.22	***/0.3	***/0.25		***/0.11	**/− 0.22		**/− 0.12		
Activated dendritic cells			**/0.14	***/− 0.24		**/-0.24	*/-0.1	*/0.11	*/− 0.1	
Resting mast cells	*/0.1			***/0.2	***/0.12		***/0.29	*/0.1	***/0.29	
Activated mast cells			*/0.1							***/− 0.2
Eosinophils				*/0.12			*/− 0.1		*/− 0.1	
Neutrophils	**/0.14		*/0.11	**/0.14				***/0.31		**/0.18

^*^p < 0.05

^**^p < 0.01

^***^p < 0.001

**Fig. 14:**
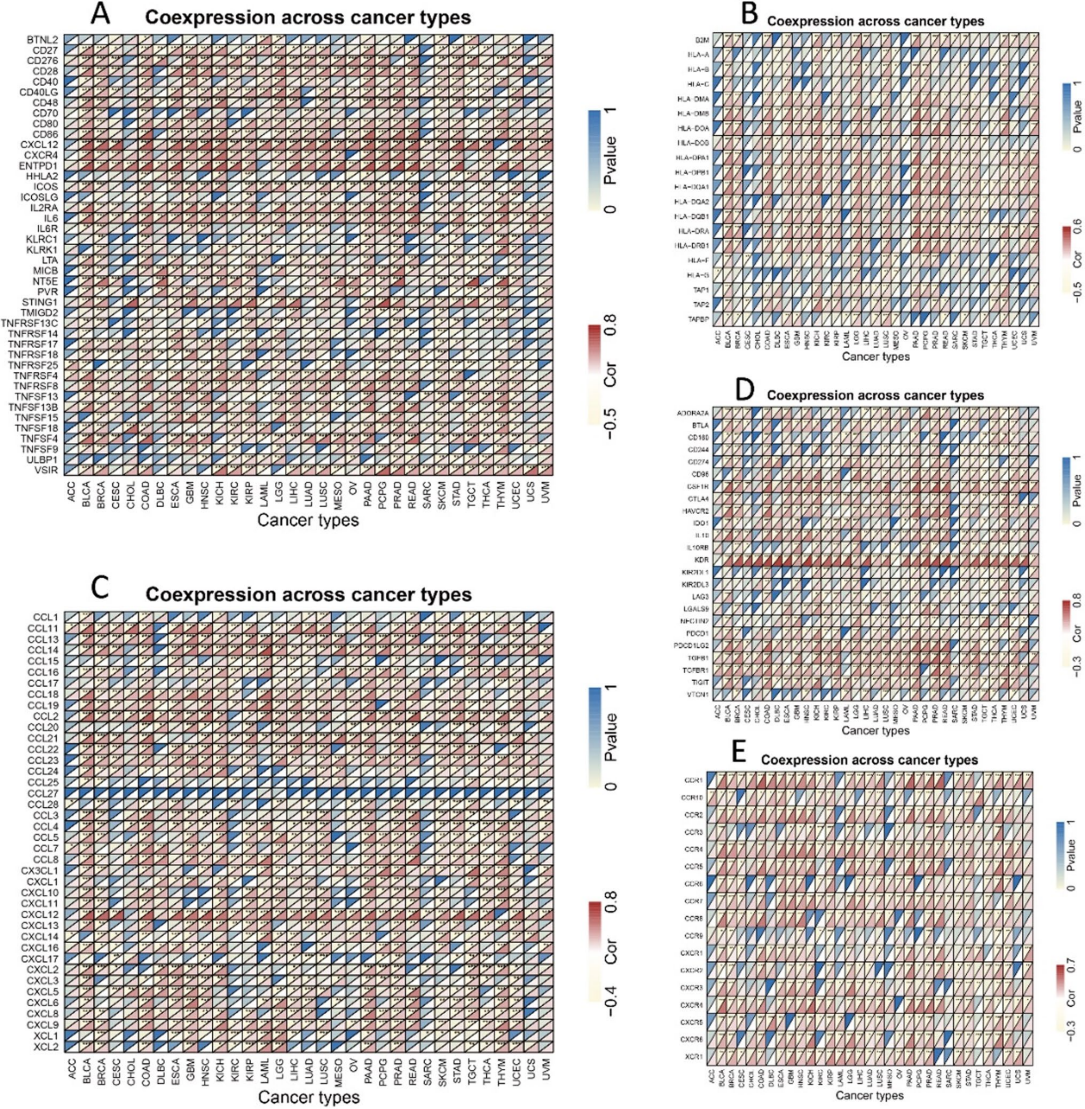
Co-expression of COL15A1 and immune-related genes. **A** Correlation with immune activation genes. **B** MYC related genes. **C** Chemokine. **D** Correlation with immunosuppressive genes. **E** Chemokine receptor. *p < 0.05, **p < 0.01, ***p < 0.001, ****p < 0.0001

Meanwhile, gene co-expression assessments were utilized to look into the association between the expression of COL15A1 and immune-related genes in 33 different tumor types. MHC, immunological activation, chemokine, chemokine, and immunosuppressive receptor proteins were among the genes studied. Almost all immune-related genes were positively linked with COL15A1 in most cancers, according to the findings, which were shown as heatmaps (Fig. 14).

### COL15A1 expression levels and CSCs

**Fig. 15:**
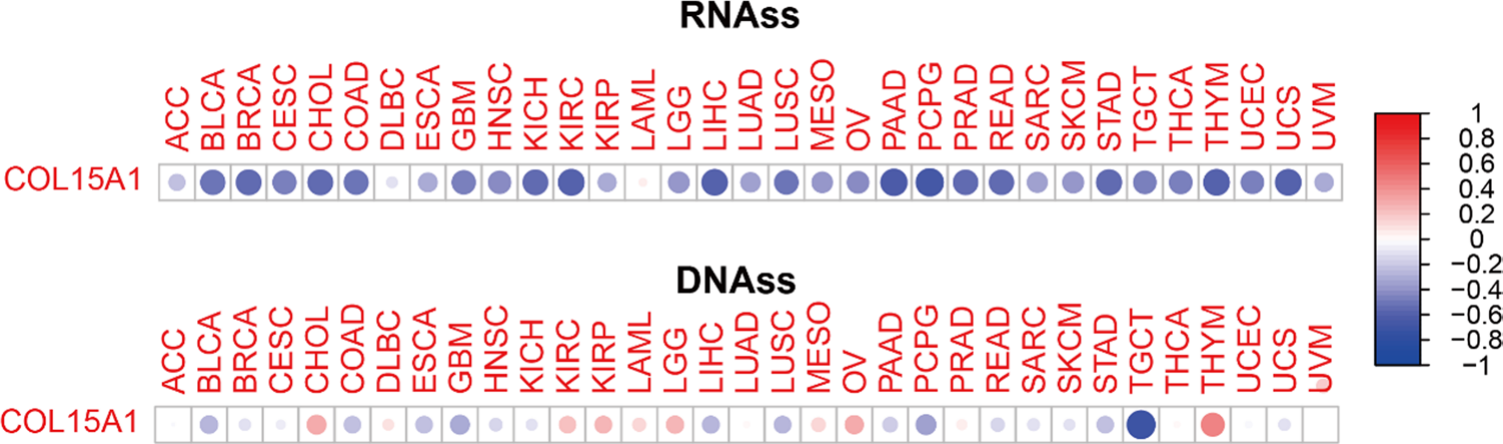
Correlation of COL15A1 expression with cancer stem cells in 33 tumors

CSCs are part of the TME, which is known to be the major factor of cancer recurrence and metastatic potential [18]. We conducted correlation analysis from two aspects of gene expression data (RNAss) and gene methylation data (DNAss) to explore the relationship between gene expression levels and CSCs (Fig. 15). The results were presented as a heatmap. For RNAss, COL15A1 expression levels were significantly negatively correlated with CSCs in most tumors (except LAML), implying that the higher the level of COL15A1 expression, the weaker the characteristics of CSCs. However, for DNAss, COL15A1 was significantly and positively correlated with CSCs in several tumors, such as CHOL, LGG, OV and THYM. Interestingly, we obtained the opposite conclusion in other tumors, including TGCT, PCPG, GBM and BLCA.

**Fig. 16:**
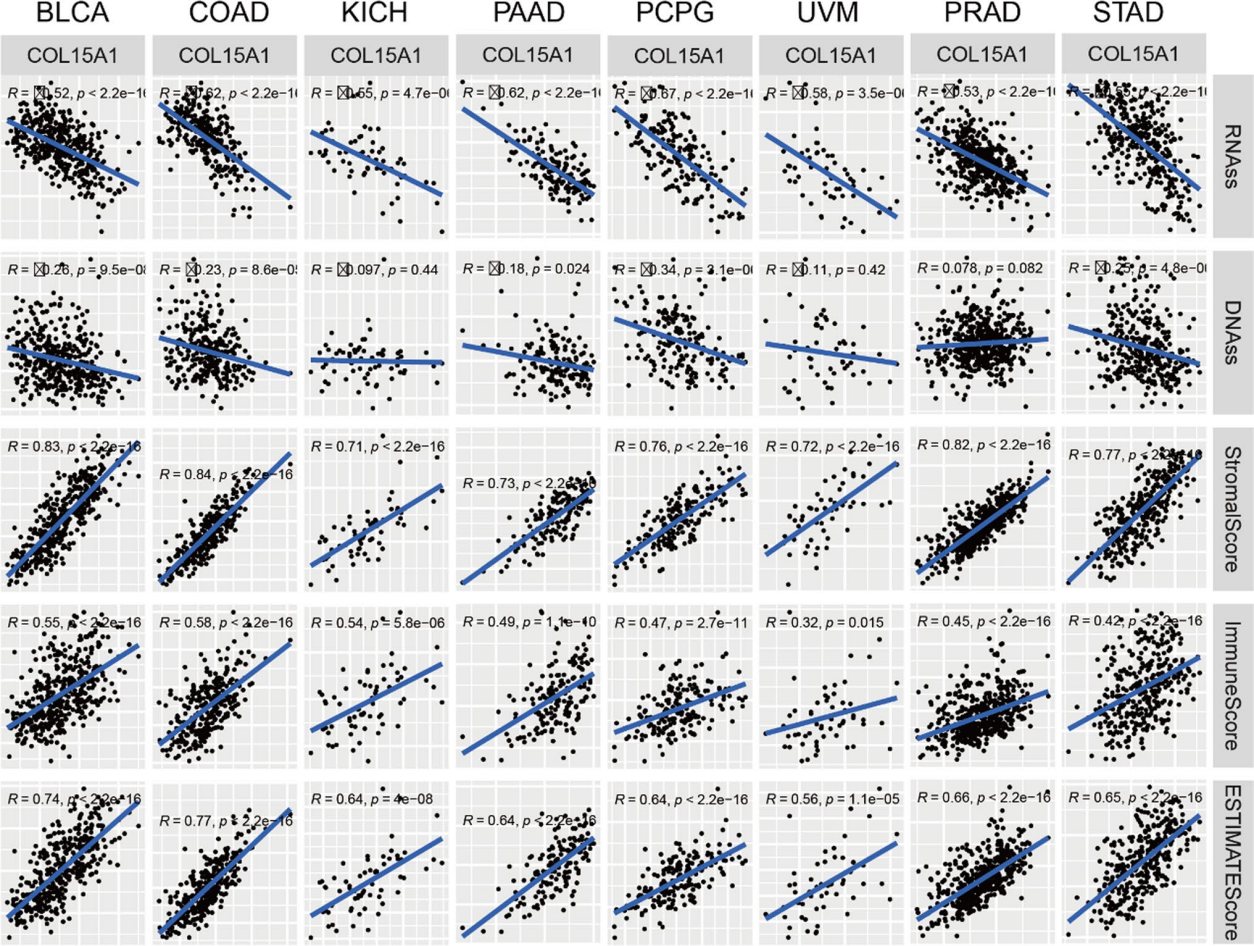
Relationship between COL15A1 expression and cancer stem cell, stromal cell scores, immune cell scores, ESTIMATE scores

In addition, five indicators, including RNAss, DNAss, stromalScore, immuneScore, ESTIMATEScore were used to analyze the COL15A1 expression levels in 33 tumors, and eight tumors were selected to present the results in Fig. 16, including BLCA, COAD, KICH, PAAD, PCPG, UVM, PRAD, STAD.

### Drug sensitivity analysis

**Fig. 17:**
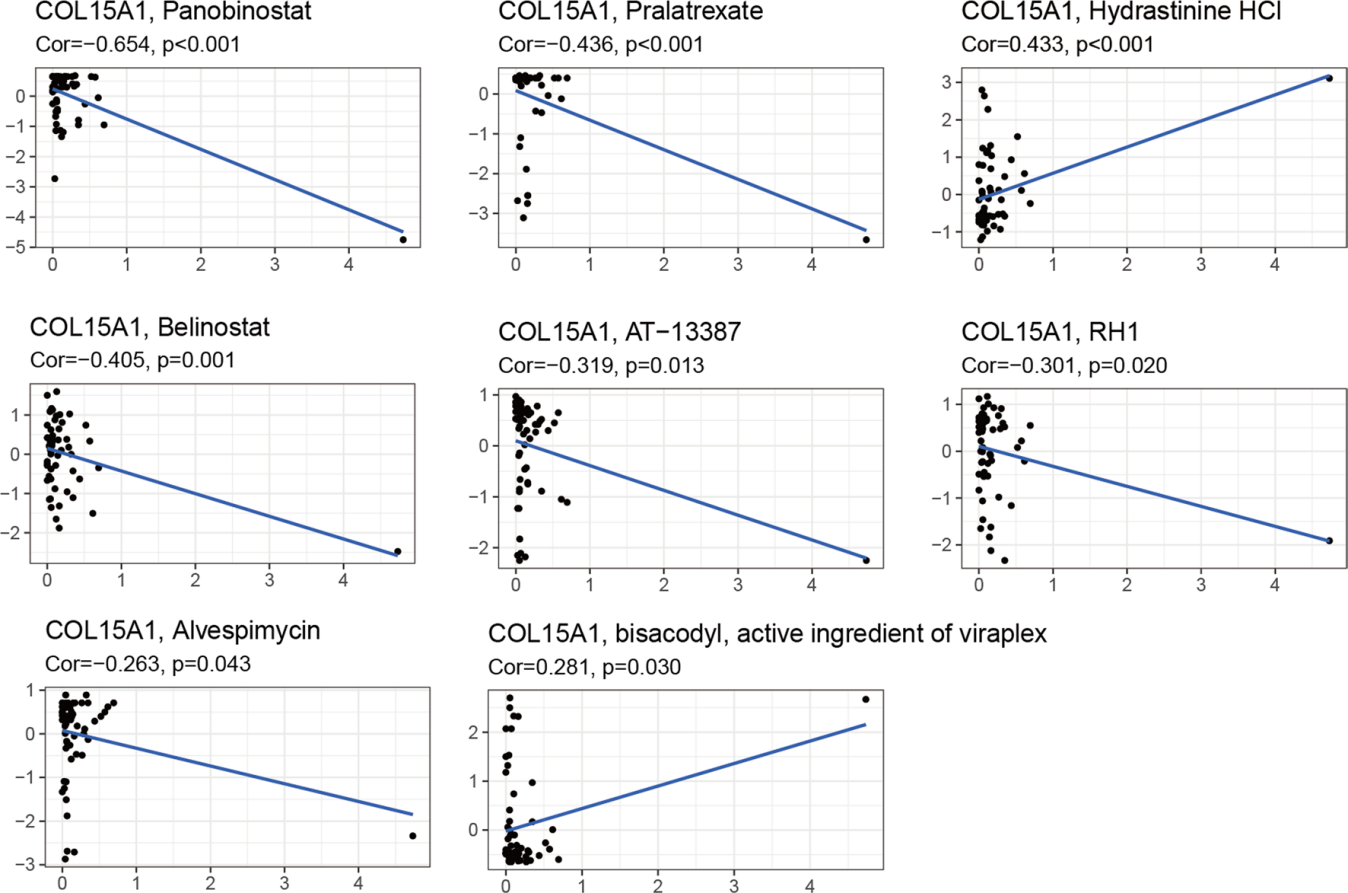
Relationship between COL15A1 expression and drug sensitivity. Horizontal coordinates represent gene expression and vertical coordinates represent drug sensitivity scores

Finding drugs with high sensitivity to tumor cells may improve the prognosis of patients. Therefore, the data from cellminer was used to explore the relationship between COL15A1 expression levels and drug sensitivity, and the eight most sensitive drugs, which may be beneficial to the tumor patient's prognosis, were selected to present the analysis results (Fig. 17). Among the eight chemotherapeutics, except Hydrastinine (Cor = 0.433, p < 0.001) and Bisacodyl (Cor = 0.281, p = 0.030), the sensitivity of most drugs to tumor cells is significantly negatively correlated with COL15A1 expression levels. The results of this analysis may provide options for clinical oncology drugs.

#### GSEA enrichment analysis

**Fig. 18:**
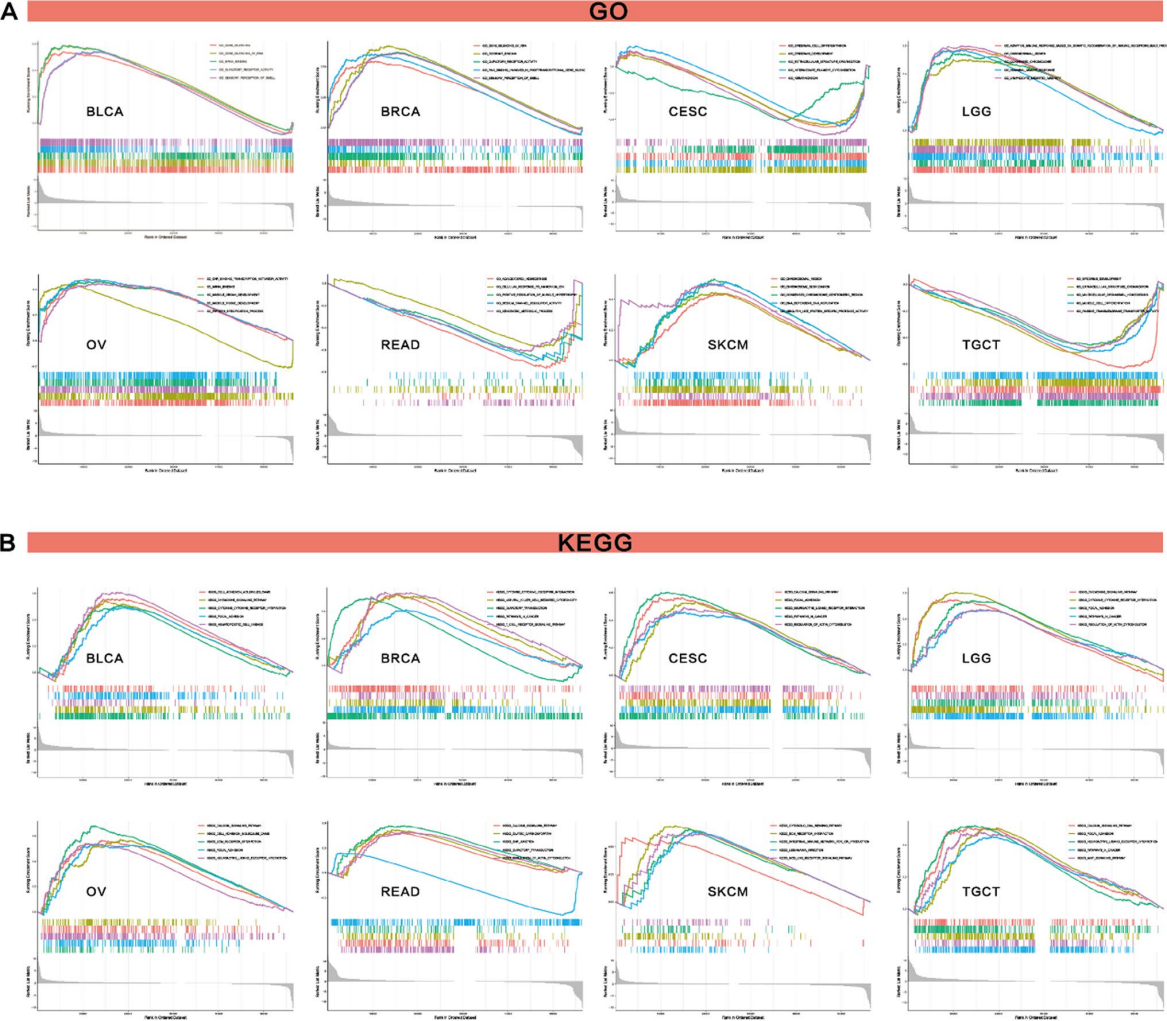
Results of GSEA. **A** GO functional annotation of COL15A1 in various cancers. **B** KEGG pathway analysis of COL15A1 in multiple cancers. Curves of different colors show different functions or pathways regulated in different cancers. Peaks on the upward curve indicate positive regulation and peaks on the downward curve indicate negative regulation

The researchers used gene set enrichment analysis (GESA) to scrutinize the biological importance of COL15A1 expression in various tumor tissues and to explain how the gene in question influenced the incidence of malignancies via functions or pathways (19). We conducted GO functional annotation and KEGG pathway assessment in 33 cancers, and we chose eight to display the results in Fig. 18. The results showed that COL15A1 negatively regulates cell transmembrane and epidermal cell differentiation in CESC, READ, and TGCT (Fig. 18A). However, GO functional analysis indicate that COL15A1 expression was positively correlated with RNA binding, RNA silencing, olfactory transduction, and other metabolic processes (Fig. 18A). In the eight tumors mentioned above, KEGG pathway analysis indicated that COL15A1 was a positive regulator of chemokines signaling pathway, cancer-related signaling pathways, and immune-related signaling pathway, such as T cell receptor signaling pathway (Fig. 18B).

#### Analysis of genes associated with COL15A1

**Fig. 19:**
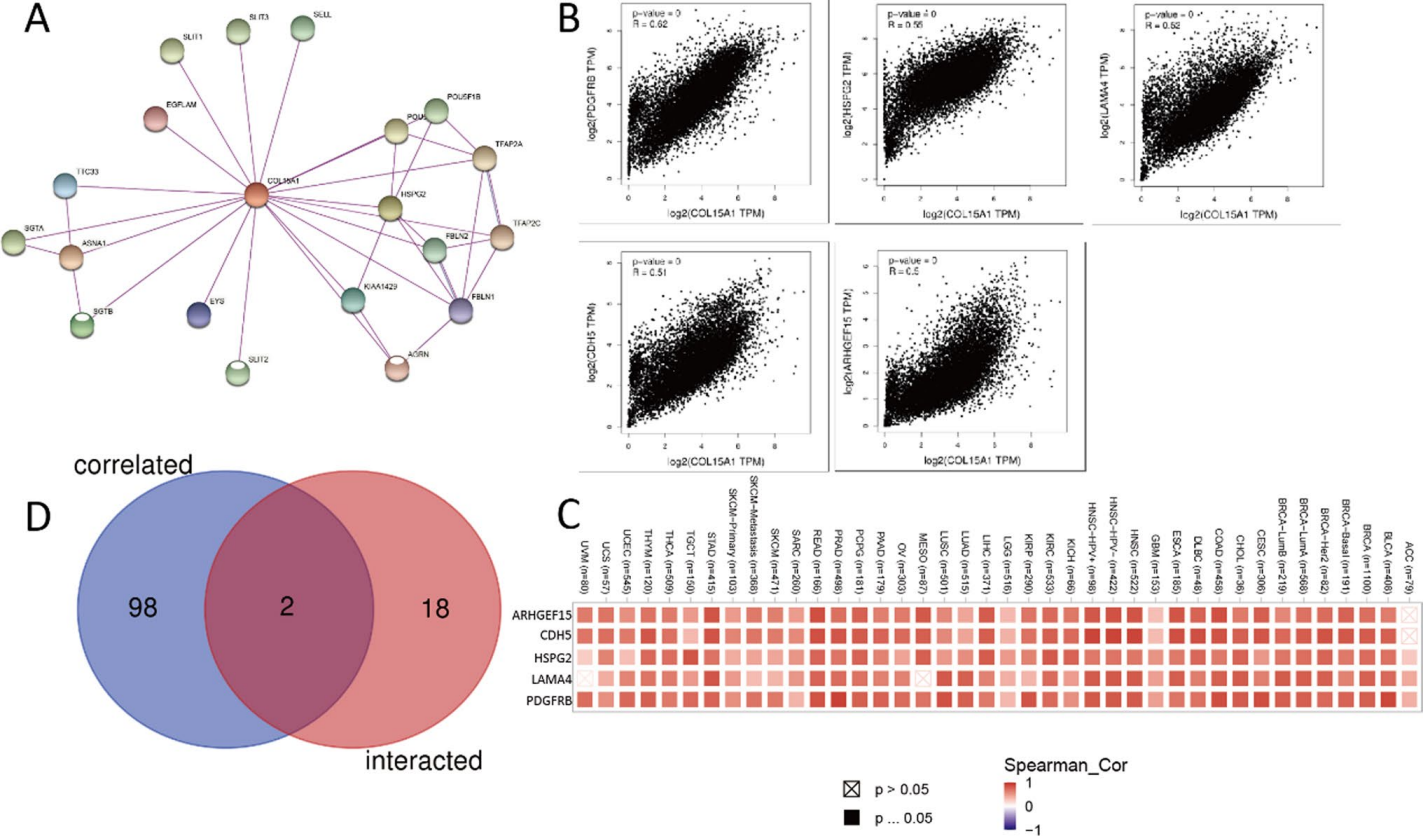
Explore similar genes for COL15A1. **A** Building protein interaction networks; **B** The five most significantly (PDGFRB, HSPG2, LAMA4, CDH5, ARHGEF15) associated genes with COL15A1; **C** Association of these five genes with COL15A1 in 33 tumors; **D** Screening for intersections of genes (HSPG2, SLIT3) in the COL15A1 correlation Top100 with protein-interacting 50 genes

The target COL15A1 binding protein and COL15A1 expression-related genes were screened to perform subsequent assessment to further scrutinize the molecular strategy of genes in carcinogenesis. Through the STRING tool (https://www.string-db.org/), we have obtained 50 COL15A1 binding proteins with experimental support, which showed the interaction network in Fig. 19A. As indicated in Fig. 19B, the COL15A1 expression is positively associated with other genes, including PDGFRB (R = 0.62), HSPG2 (R = 0.55), LAMA4 (R = 0.52), CDH5 (R = 0.51), ARHGEF15 (R = 0.5). The corresponding heatmap data also proves the conclusion that the expression of COL15A1 is significantly positively correlated with these five genes in most tumors (Fig. 19C). Furthermore, we obtained the 100 genes most relevant to COL15A1 expression through the TCGA data of the GEPIA2 tool. The genes SLIT3 and HSPG2 were discovered to be common members of the two groups after an intersection analysis of their genes (Fig. 19D).

**Fig. 20:**
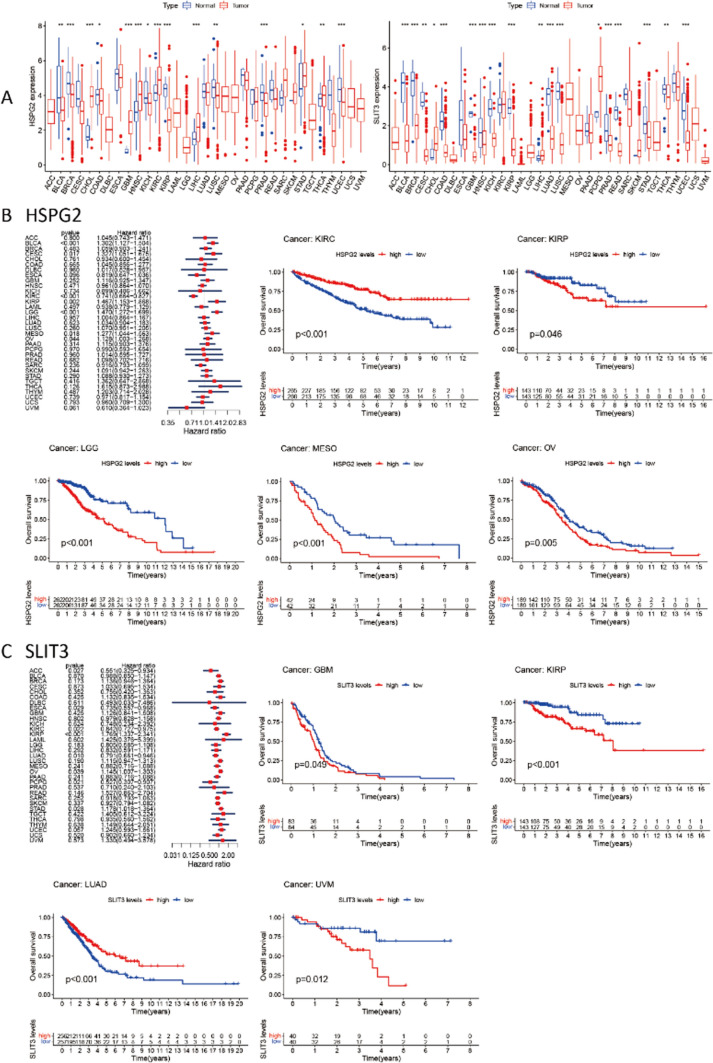
Differential expression of HSPG2, SLIT3 and tumor prognosis. **A** Expression of two genes in tumor and normal tissues; **B** COX analysis of HSPG2 and overall survival time in days (OS); **C** COX analysis of SLIT3 and overall survival time in days (OS)

Then, using the prior data, we analyzed the differences in the expression of these two genes in 33 tumors (Fig. 20A). The results revealed that the expression of these two genes in normal and malignant tissues differs significantly. Subsequently, overall survival (OS) analysis and COX analysis were completed by the method which was used to evaluate patient prognosis (Fig. 20B, C). As shown in Fig. 20, although the COX analysis is not statistically significant, the expression level of genes significantly affects the survival of cases in some tumors, such as KIRC, LGG, KIRP, and LUAD. This may mean that COL15A1 and these two genes interfere with the occurrence of tumors.

#### Analysis of COL15A1 gene mutation

**Fig. 21:**
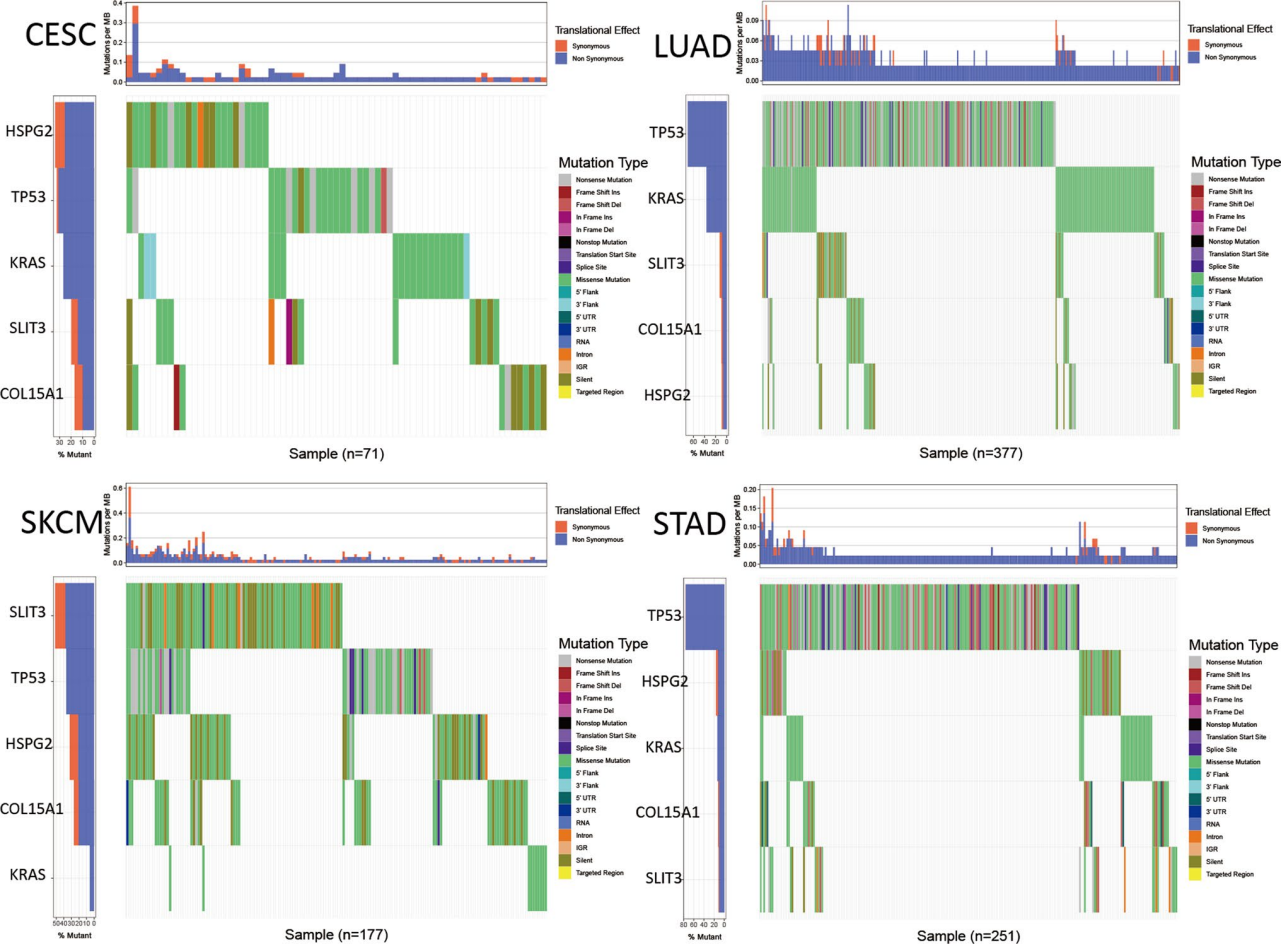
Single nucleotide polymorphism (SNP) analysis of COL15A1 in multiple tumors

**Fig. 22:**
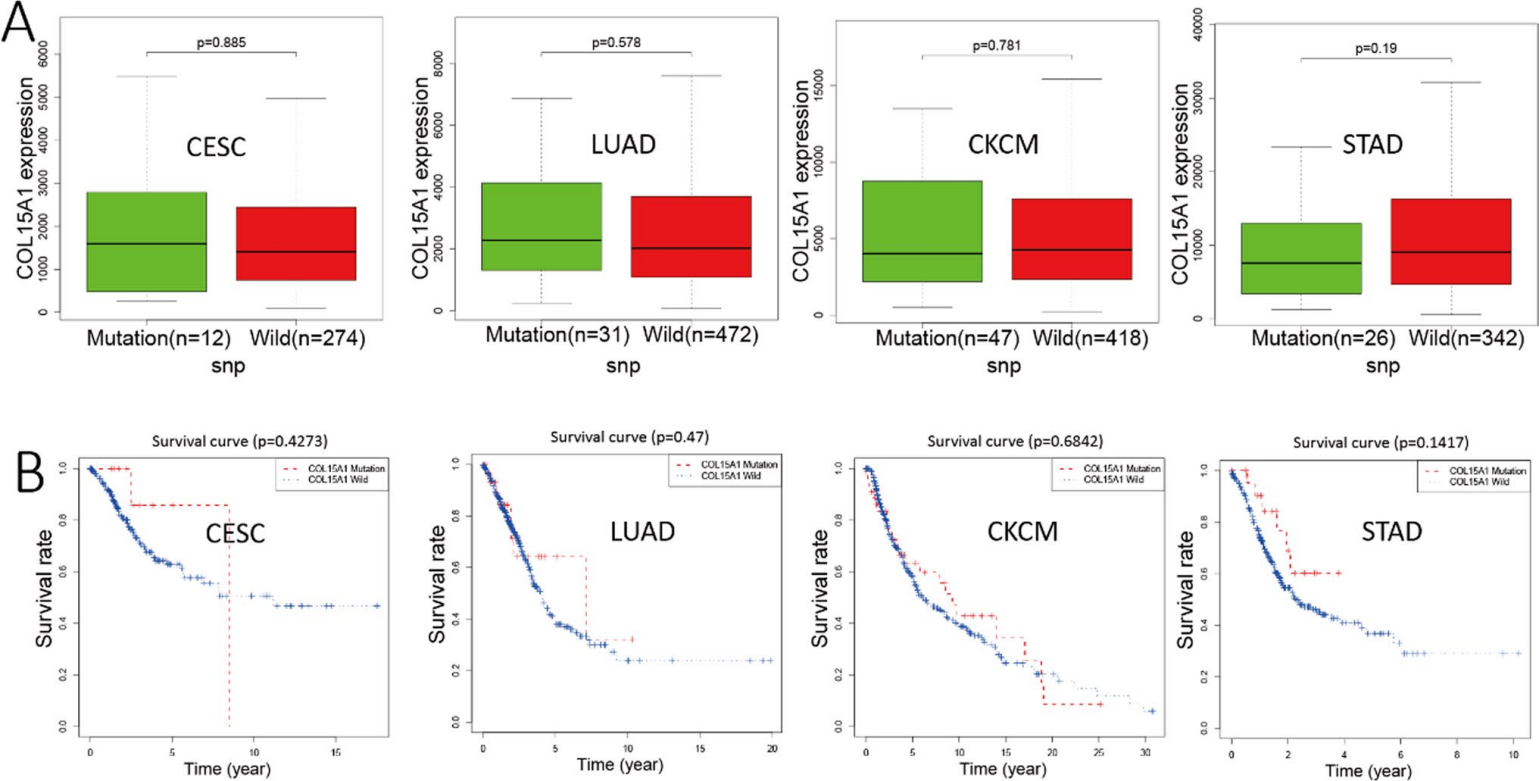
SNP expression and survival analysis of COL15A1 in different tumors

Single nucleotide polymorphism (SNP) is DNA sequence polymorphisms caused by variants in individual nucleotides at the genomic level that promote tumorigenesis [19, 20]. We studied the variation of genes in all samples of 33 tumors and selected four tumors to present (Fig. 21), including CESC, LUAD, SKCM, STAD. Taking TP53 and KRAS as controls, COL15A1 has significant variation in tumor samples, suggesting us that the COL15A1 mutation may promote tumorigenesis. In addition, we analyzed the COL15A1 expression levels in mutant and wild-type samples and found that there was no significant correlation between them (Fig. 22A, P > 0.05). Similarly, COL15A1 expression and patient’s prognostic survival analysis were not statistically significant in wild-type and mutant samples (Fig. 22B, P > 0.05). These results suggest that the nucleotide variation of COL15A1 seems to have little effect on tumorigenesis.

#### COL15A1 inhibits the growth of HCC

**Fig. 23:**
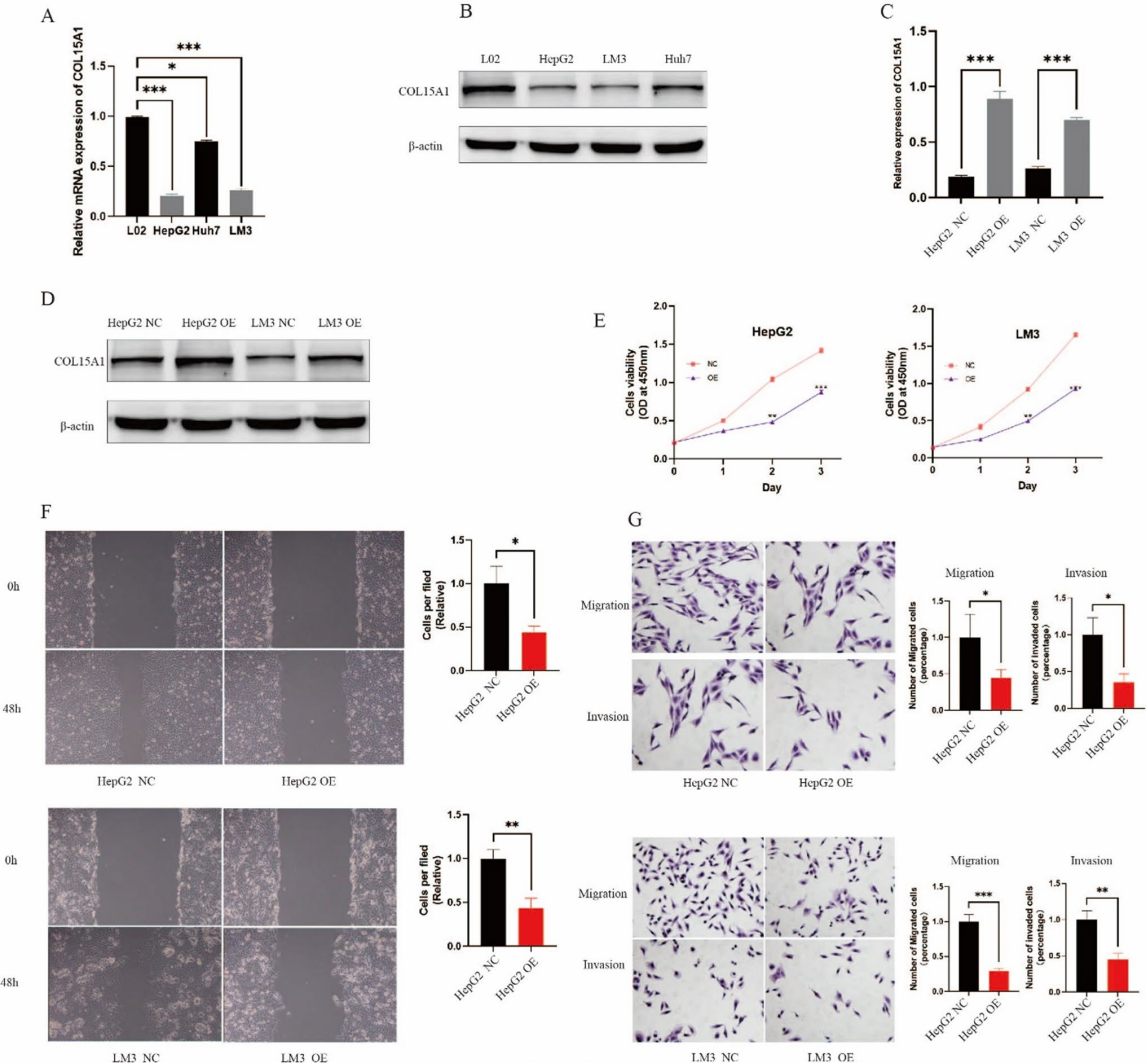
COL15A1 inhibits hepatocellular carcinoma cell progression. **A**, **B** The expression level of COL15A1 in different cells was observed by qRT-PCR and Western blot. **C**, **D** The overexpression efficiency (OE) of COL15A1 was verified by qRT-PCR and Western blot. **E** CCK8 assay was performed to detect the effect of COL15A1 on hepatocellular carcinoma cell proliferation. **F** Wound healing assay was utilised to observe the the effect of COL15A1 on the migration of hepatocellular carcinoma cells. **G** The effects of COL15A1 on migration and invasion of hepatocellular carcinoma cells were observed by Transwell assay

To investigate the effect of COL15A1 on tumor progression, we selected hepatocellular carcinoma cells to verify its effect on tumor cell progression. With qRT-PCR and Western blot we found that the expression of COL15A1 in hepatocellular carcinoma tissues was lower than that in normal liver tissues (Fig. 23A, B), and we selected types of hepatocellular carcinoma cells (HepG2 and LM3) for subsequent work. Subsequently, we overexpressed COL15A1 in hepatocellular carcinoma cells and detected the expression level of COL15A1 in the tumor cells by qRT-PCR and Western blot (Fig. 23C, D). CCK8 assay was employed to observe the effect of COL15A1 on the proliferative viability of hepatocellular carcinoma cells, and the results showed that high expression of COL15A1 significantly inhibited the proliferation of tumor cells (Fig. 23E). Meanwhile, Transwell assay showed that overexpression of COL15A1 inhibited the migration and invasion of hepatocellular carcinoma cells (Fig. 23F). Similarly, the inhibition of hepatocellular carcinoma cell migration by high expression of COL15A1 compared to the control group could be observed in the wound healing assay (Fig. 23G).

## Discussion

Whether COL15A1 plays a role in the pathogenesis of different tumors through some common molecular mechanisms is unclear. Through the literature search, we were unable to retrieve any articles on pan-cancer analysis of COL15A1 from the perspective of overall tumors. Therefore, we performed a comprehensive evaluation of COL15A1 in 33 tumors using the data of TCGA and UCSC.

In this study, COL15A1 was highly expressed in 13 tumors (Fig. 1), including LIHC and STAD. This is consistent with the findings of the previously reported studies [21, 22] and was further confirmed by IHC at the protein level. COL15A1 has been reported as a potential prognostic marker [23, 24]. In previous studies, high expression of COL15A1 promoted gastric cancer progression at the protein level by affecting lipid metabolism [22], this is in good agreement with the findings of our research. However, the COL15A1 expression has been reported to be substantially greater in hepatocellular carcinoma compared to normal liver tissue, thus leading to poor prognosis [21]. In our study, high COL15A1 expression indicates a longer survival time in LIHC. Because our sample was more of an in situ tumor than a metastatic tumor, this variation may be different for tumor samples. Furthermore, the role of COL15A1 is different in various cancers due to the biodiversity. As described in our study, COL15A1 is a risk factor in ACC and a protective factor in READ (Fig. 3A). This difference was also validated by the expression levels of COL15A1 in distinct tumor tissues. COL15A1 expression, for example, was considerably higher in ACC than in LGG. Low COL15A1 expression is related to poor prognosis in KIRC and SARC, regarding the findings of the KM survival assessment (Fig. 3). In PCPG, greater COL15A1 expression is associated with a prolonged survival time (Fig. 3) [9]. In KIRP and MESO, however, increased COL15A1 expression is linked to a poor prognosis (Fig. 4), suggesting that COL15A1 could be a risk factor for individuals with these cancers. These findings suggest that COL15A1 has many roles in the progression of malignancies and is a promising predictor for malignant prognosis.

Moreover, we discovered a considerable correlation between COL15A1 expression and tumor stage in certain cancer types, including HNSC, KIRC, TGCT, and KIRP (Fig. 7). This correlation was more pronounced between stage I and II or stage I and III. We also found that gene expression was associated with age in some tumor types, such as BLCA, BRCA, LIHC, SARC, THYM, and UCEC. COL15A1 expression was lesser in younger cases with BLCA and THYM. Among the other four tumors, COL15A1 expression was lesser in younger cases. These findings could have ramifications for the selection of immunotherapy regimens for individuals of various ages and disease stages.

TMB is a promising pan-cancer prognostic marker that can also be used to direct immunological precision treatment [25, 26]. TMB as a biomarker, for example, has been found in prior trials to boost immunotherapy efficacy in breast cancer [27]. TMB was also useful in predicting the prognosis of cancer patients, particularly those with LUAD and HNSC who were receiving immunotherapy [28]. MSI is a key biomarker for immune checkpoint inhibitors (ICI), as well as a diagnostic characteristic for a variety of malignancies [29, 30]. Furthermore, in colorectal cancer, high-frequency MSI is an independent predictor of prognosis and clinical profiles [30]. MSI and TMB expression levels were found to be significantly correlated in various tumor types (Fig. 8), including THYM and COAD. This could mean that COL15A1 expression levels can alter the TMB and MSI of the tumor, thereby impairing patient response to immune checkpoint inhibitor therapy and resulting in poor tumor prognosis. Furthermore, we discovered that COL15A1 expression levels were substantially linked with MMR in the majority of cancers, including DLBC and PAAD. In tumors where COL15A1 expression is positively linked with TMB, we infer that tumors with considerable COL15A1 expression, as well as considerable MSI and TMB, may lead to poor prognosis following the ICI treatment.

TME levels are linked to gene expression levels. Tumor-infiltrating immune cells can be employed as a marker to measure tumor cell responsiveness to immunotherapy and impact clinical outcomes as part of the TME. We couldn't find any beneficial information in the prior literature because there had been few investigations on the association between COL15A1 and tumor immune cells. COL15A1 expression was favorably linked with stromal cells and immune cells in TME of different cancers, according to a research of ESTIMATE scores (Fig. 10). CAFs are also important components of stromal cells, and they've been linked to poor prognosis, treatment resistance, and disease recurrence in a variety of malignancies. Our findings revealed that COL15A1 expression was substantially linked with CAFs, implying that COL15A1 may influence tumor growth in the TME through modulating immunological, stromal, and CAF cells. Furthermore, this study shows that COL15A1 expression takes part in the biological procedures of immune-related molecular entities and immune cells in most tumors, and it confirms that COL15A1 expression is intimately involved in the biological procedures of immune-related molecules and immune cells in the majority of tumors. COL15A1 was shown to be co-expressed with genes encoding MHC, immunological activation, immunosuppression, chemokine receptor proteins, and chemokines, according to our findings (Fig. 14). These findings imply that COL15A1 expression is linked to tumor cell immune infiltration, has an impact on patient prognosis, and could be used to design novel immunosuppressants. Furthermore, drug sensitivity analysis revealed numerous medicines that had a substantial correlation with COL15A1 expression (Fig. 17), suggesting that these drugs could be useful in oncology treatment.

Furthermore, our enrichment analysis suggests that COL15A1 may participate in a wide range of metabolic pathways and biosynthesis to influence tumor etiology or pathogenesis through certain pathways, including regulation of immune response signaling, sensory-perceptual signaling, cytokine, and chemokine pathways, and RNA metabolic pathways. These findings backed up our gene enrichment study and cleared the path for more research into the molecular function of COL15A1. We discovered many genes (SLIT3 and HSPG2) which were co-expressed with COL15A1 across various cancers and tissues using STRING and GEPIA2. Through COX analysis and survival analysis, we discovered that these two genes were differently expressed in 33 cancers and affected tumor prognosis, and we concluded that these two genes operated synergistically with COL15A1 to promote tumor growth and modify patient prognosis. There was no statistically significant link between SNP expression and tumor prognosis with COL15A1 as a kind of gene mutation in 33 tumor types (Figs. 21, 22). This isn't to say that other types of gene alterations can’t cause COL15A1 to cause poor tumor prognosis.

To conclude, our pan-cancer analysis divulged substantial discrepancies in COL15A1 expression across normal and malignant tissues, and also a link between COL15A1 expression and clinical prognosis. The findings of the current work imply that varied levels of COL15A1 expression impose diverse prognostic outcomes on malignancies and that this gene can act as an independent prognostic factor for various tumors. In addition, immune infiltration assessment and COL15A1-related gene enrichment assessment suggest that COL15A1 may influence tumor immunity, DNA repair, or cell cycle in malignancies. However, because we conducted a comprehensive pan-cancer analysis, more investigation is essential to understand the precise molecular performance of COL15A1 in carcinogenesis. Furthermore, in diverse cancer types, COL15A1 expression levels were substantially linked with TMB, MSI, and immune cell infiltration. The result could facilitate clarifying the precise function of COL15A1 in carcinogenesis and serve as a benchmark for future precision and targeted immunotherapy.

## Data Availability

All data in the article were from publicly available databases, including TCGA (https://portal.gdc.cancer.gov/), UCSC Xena (http://xena.ucsc.edu/), HPA (https://www.proteinatlas.org/), TIMER2.0 (http://timer.cistrome.org/), CellMiner (https://discover.nci.nih.gov/cellminer/home.do). All the datasets were open access datasets.

## Abbreviations

ACC: Adrenocortical carcinoma
BLCA: Bladder urothelial carcinoma
BRCA: Breast invasive carcinoma
CAFs: Cancer fibroblasts
CESC: Cervical squamous cell carcinoma and endocervical adenocarcinoma
CHOL: Cholangiocarcinoma
COAD: Colon adenocarcinoma
COL15A1: Collagen, type XV, alpha 1
CSCs: Cancer stem cells
DFI: Disease-free interval
DLBC: Lymphoid neoplasm diffuse large b-cell lymphoma
DSS: Disease-specific survival
ESCA: Esophageal carcinoma
GBM: Glioblastoma multiforme
GO: Gene Ontology
GSEA: Gene set enrichment analysis
HNSC: Head and neck squamous cell carcinoma
KEGG: Kyoto Encyclopedia of Genes and Genomes
KICH: Kidney chromophobe
KIRC: Kidney renal clear cell carcinoma
KIRP: Kidney renal papillary cell carcinoma
KM: Kaplan–Meier
LAML: Acute myeloid leukemia
LGG: Brain lower grade glioma
LIHC: Liver hepatocellular carcinoma
LUAD: Lung adenocarcinoma
LUSC: Lung squamous cell carcinoma
MESO: Mesothelioma
MHC: Major histocompatibility complex
MMR: Mismatch repair
MSI: Microsatellite instability
OS: Overall survival
OV: Ovarian serous cystadenocarcinoma
PAAD: Pancreatic adenocarcinoma
PCPG: Pheochromocytoma and paraganglioma
PFI: Progression-free interval
PRAD: Prostate adenocarcinoma
READ: Rectum adenocarcinoma
SARC: Sarcoma
SKCM: Skin cutaneous melanoma
SNP: Single nucleotide polymorphism
STAD: Stomach adenocarcinoma
TGCT: Testicular germ cell tumors
THCA: Thyroid carcinoma
THYM: Thymoma
TMB: Tumor mutational burden
TME: Tumor microenvironment
UCEC: Uterine Corpus Endometrial Carcinoma
UVM: Uveal Melanoma
